# Diversity in Aβ deposit morphology and secondary proteome insolubility across models of Alzheimer-type amyloidosis

**DOI:** 10.1186/s40478-020-00911-y

**Published:** 2020-04-06

**Authors:** Guilian Xu, Susan E. Fromholt, Paramita Chakrabarty, Fanchao Zhu, Xuefei Liu, Michael C. Pace, Jin Koh, Todd E. Golde, Yona Levites, Jada Lewis, David R. Borchelt

**Affiliations:** 1 Department of Neuroscience, Center for Translational Research in Neurodegenerative Disease, McKnight Brain Institute, College of Medicine, University of Florida, Gainesville, FL 32610 USA; 2 The Interdisciplinary Center for Biotechnology Research (ICBR), University of Florida, Gainesville, FL 32610 USA; 3 SantaFe Healthcare Alzheimer’s Disease Research Center, Gainesville, FL USA

## Abstract

A hallmark pathology of Alzheimer’s disease (AD) is the formation of amyloid β (Aβ) deposits that exhibit diverse localization and morphologies, ranging from diffuse to cored-neuritic deposits in brain parenchyma, with cerebral vascular deposition in leptomeningeal and parenchymal compartments. Most AD brains exhibit the full spectrum of pathologic Aβ morphologies. In the course of studies to model AD amyloidosis, we have generated multiple transgenic mouse models that vary in the nature of the transgene constructs that are expressed; including the species origin of Aβ peptides, the levels and length of Aβ that is deposited, and whether mutant presenilin 1 (PS1) is co-expressed. These models recapitulate features of human AD amyloidosis, but interestingly some models can produce pathology in which one type of Aβ morphology dominates. In prior studies of mice that primarily develop cored-neuritic deposits, we determined that Aβ deposition is associated with changes in cytosolic protein solubility in which a subset of proteins become detergent-insoluble, indicative of secondary proteome instability. Here, we survey changes in cytosolic protein solubility across seven different transgenic mouse models that exhibit a range of Aβ deposit morphologies. We find a surprisingly diverse range of changes in proteome solubility across these models. Mice that deposit human Aβ40 and Aβ42 in cored-neuritic plaques had the most robust changes in proteome solubility. Insoluble cytosolic proteins were also detected in the brains of mice that develop diffuse Aβ42 deposits but to a lesser extent. Notably, mice with cored deposits containing only Aβ42 had relatively few proteins that became detergent-insoluble. Our data provide new insight into the diversity of biological effects that can be attributed to different types of Aβ pathology and support the view that fibrillar cored-neuritic plaque pathology is the more disruptive Aβ pathology in the Alzheimer’s cascade.

## Introduction

Alzheimer’s disease (AD) is the most common form of dementia in aged populations [10]. The two primary pathological hallmarks of the AD brain are abnormal extracellular deposits of β-amyloid (Aβ) peptide and intracellular neurofibrillary tau tangles (NFTs) [17, 22]. The deposition of Aβ peptides in the form of amyloid plaques is a critical early step in the disease process that is hypothesized to trigger a complex pathological cascade that ultimately leads to the development of clinical dementia [3, 32] . Indeed, familial early-onset AD (FAD) is caused by altered production of Aβ peptides due to mutations in the amyloid precursor protein (APP) or presenilin (PS) 1 and PS2, which are components of one of the endoproteinases that cleaves APP to produce Aβ peptides [22]. Further, there is strong evidence that the genetic risk for AD that has been associated with polymorphisms in both apoliprotein E (APOE) and clusterin (CLU) is at least partially attributable to effects of these proteins on Aβ deposition [2, 8, 11, 24]. Collectively, these genetic studies indicate that the deposition of Aβ can be a powerful trigger in the pathogenesis of AD-related dementia.

Aβ deposits in the parenchyma of human brain have been classified as diffuse, fibrillar or dense-cored [9, 37, 39]. Fibrillar plaques have a central mass of Aβ with spoke-like extensions leading to a confluent outer rim. Dense-cored plaques have a compact core of Aβ surrounded by a less dense perimeter of Aβ. Diffuse plaques lack an identifiable substructure and are homogenously labeled by anti-Aβ antibodies. Both fibrillar and dense core plaques are strongly stained by Congo red or Thioflavin S (ThioS) whereas diffuse plaques are Congo Red negative and weakly positive for ThioS or negative. Both dense-cored and fibrillar plaques are commonly large, spherical structures surrounded by dystrophic neurites, reactive astrocytes and microglia. These types of plaques are referred to as neuritic plaques (NPs) and a semi-quantitative assessment of this pathology is used in the pathological diagnosis of AD [12, 30]. By contrast, diffuse Aβ deposits are a normal feature of the aged brain and not considered pathognomonic for AD [12, 20, 30]. How these different types of Aβ pathology arise and how they are related to each other is unclear, and our understanding of the biological consequences of these different types of pathology on CNS homeostasis is limited.

In prior studies of the APPswe/PS1dE9 model of Alzheimer-type amyloidosis, we observed age-dependent changes in the solubility of the proteome such that cytosolic brain proteins became over-represented in SDS-insoluble extracts [34, 42]. A subset of the proteins identified in the APPswe/PS1dE9 model were also identified as losing solubility in mice that model neurofibrillary tangle (NFT) pathology, superoxide dismutase 1 (SOD1) pathology, and α-synuclein pathology [34]. Importantly, it did not appear that the changes in solubility of any one protein were sufficient to lower function, leading us to view the observed changes in protein solubility as a biomarker of general proteostasis dysfunction [34]. The proteins we identify as insoluble may be the result of reduced capacity to fold newly-made proteins or to lowered efficiency in degrading proteins that have failed to achieve native conformations [40]. Here, we analyze changes in proteome solubility across a panel of seven different mouse models of amyloidosis depositing human or mouse Aβ. These models exhibit transgene-dependent variations in the neuropathological features ranging from predominately cored-neuritic Aβ deposits to predominantly diffuse deposits [13, 14, 41]. We found that mice exhibiting cored-neuritic deposits, containing human Aβ40 and Aβ42, had the highest numbers of cytosolic proteins with altered detergent solubility. Mice in which diffuse Aβ deposits predominated, primarily composed of Aβ42, had relatively few proteins that showed altered detergent solubility. These data are consistent with the notion that Alzheimer-type amyloidosis can be generated by different conformers of Aβ, which in-turn produce distinct biologic consequences on CNS function.

## Materials and methods

### Transgenic animals

To model amyloidosis, we used seven different transgenic mouse models (Table 1). Most of the lines of transgenic mice used in this study have been described previously including the following; mice that express mouse APP with a humanized Aβ sequence that is co-expressed with human mutant PS1 [human/mouse APPswe/PS1dE9 mice (PrP.HuAβ/PS1) [13, 15]; mice expressing mouse APP with mutant human PS1 to deposit mouse Aβ [PrP.MoAβ (Line D-943) [41]); mice expressing human/mouse APPswe/ind using the MoPrP. Xho vector that was co-injected with a vector to express eGFP in skin (PrP.APPsi [41]). All three of these lines of mice were maintained on a hybrid strain background of C3H/HeJ x C57BL/6 J, following a breeding scheme in which transgene positive males were breed to F1 B6/C3 mice. We also used two lines of mice that used vectors for tetracycline regulated expression to produce transgenics including; mice expressing a humanized mouse APP gene with familial AD mutations (swe/ind) [Tet.HuAβ mice (Line 107xtTA) [14]); and mice expressing a mouse APPswe/ind gene that was co-injected with a gene construct to express eGFP in skin (Tet.MoAβ [41])]. The breeding scheme used to produce Tet.HuAβ mice involved maintaining bigenic breeding stock males as congenics on the C57BL/6 J strain on a diet that included doxycycline (Dox) to suppress APP expression, feeding the breeding females (FVB/NJ strain) Dox-containing diets until offspring were weaned at ~ 28 days old as described previously [27]. Some cohorts of these mice were administered Dox-containing food again for either 1 week or 4 weeks before harvesting at 13 months of age. The Tet.MoAβ mice were generated on the FVB/NJ background and bred to B6.Cg mice harboring the CamKII-tTA transgene as previously described [41]. Mice that are homozygous for the BriAβ42 (Bri42) transgene were generated by inbreeding previously decribed lines of mice [26]. We created a new line of inducible Bri42 mice by inserting the Bri42 transgene into the TetPrP. Xho vector previously described by Jankowsky et al. [14]. Founder lines were generated in the FVB/NJ strain and screened by crossing Tet.Bri42 mice with mice harboring the CaMKII-tTA transgene (B6 congenic) to drive expression from the TetPrP. Xho vector, following a strategy previously described in generating mice with inducible expression of mutant APP [14]. A line of mice was identified that expressed high levels of Bri42 and developed Aβ deposits by 2 months of age (Table 1). All Tet.Bri42 mice used in this study were F1 hybrids on the FVB/NJ and C57BL/6 J background strains of mice.

**Table 1:** Attributes of the mouse models used in this study

Model Designation	Other names	Vector	Transgenes	Aβ species	Onset Aβ deposition (mo)	Neuropath features of aged animals	Reference
**Bri42 Homo**	HBri	Mo.PrP	Bri-HuAβ42 (homozygous)	Hu	~ 3	Diffuse > Cored, Thio +	[26]
**Tet.Bri42**	Bri42 x tTA	Tet-PrP CamKII-tTA	Bri-HuAβ42, tTA	Hu	< 2	Diffuse ≈ Cored, Thio +	Current study
**PrP.HuAβ/** **PS1**	APPswe/PS1dE9 Line 85	Mo.PrP	m/hAPPswe+PS1dE9	Hu	~ 6	Cored> > diffuse, Thio + with CAA	[13]
**PrP.MoAβ/** **PS1**	MoAPPswe/PS1dE9, Line D-943	Mo.PrP	moAPPswe+PS1dE9	Mo	~ 14	Cored Thio + with CAA	[41]
**PrP.APPsi**	MHSI-695, PrP.HuAβ (GFP)	Mo.PrP	m/hAPPswe/ind, eGFP	Hu	~ 12	Diffuse > > Cored, Thio +	[41]
**Tet.MoAβ**	Tet-moAPPsi, moAPPxtTA	Tet-PrP CamKII-tTA	moAPPswe/ind, tTA, eGFP	Mo	~ 13	Diffuse	[41]
**Tet.HuAβ**	Tet.APPsi, 107 x tTA	Tet-PrP CamKII-tTA	m/hAPPswe/ind, tTA	Hu	~ 3	Cored>diffuse, Thio +	[14]

All mice requiring genotyping were marked by tail tattoo and genotyped via PCR of tail DNA, following protocols similar to those previously described [13]. Homozygous Bri42 mice did not require genotyping and PrP.APPsi mice were genotyped by visualizing GFP expression, which was made possible by co-injection of a transgene for GFP that is expressed in the skin [41]. All animals were housed up to 5 per cage with unlimited access to food and water with a 14-h light and 10-h dark cycle. All experiments involving mice were approved by the University of Florida Institutional Animal Care and Use Committee (IACUC) and conducted in accordance with NIH guidelines.

### Tissue collection

Mice were anesthetized with isoflurane and perfused transcardially with 20 ml of cold PBS. For the purposes of liquid chromatography tandem mass spectrometry (LC-MS/MS) experiments, brains were quickly excised and the forebrains dissected and briefly kept in centrifuge tubes on ice before homogenization and detergent extraction as described previously [42]. For the purposes of histology and ELISA, brains were cut sagittally through the midline, and one hemi-brain was immersion-fixed in 4% paraformaldehyde in PBS (pH 7.5) for ~ 48 h at 4 °C followed by processing and paraffin embedding. The other hemibrain was snap frozen on dry-ice and then stored at -80 °C until it was thawed and homogenized in preparation for ELISA measurements of Aβ peptide levels.

### Histology and immunochemistry

Paraffin sections (5 μm) were used for all the histology and immunochemistry studies. The methods used in Campbell-Switzer silver staining [38], ThioS staining and Congo Red staining have been described previously [41]. Immunochemistry followed standard protocols described previously [41]. The primary antibodies used for immunostaining were 4G8 (1:250, mouse monoclonal, Cat. SIG-39220, Covance/BioLegend, San Diego, CA), pan Aβ monoclonal antibody MM27–33.1.1 (1:500, Technology #2008–121, T. Golde, Mayo Clinic Ventures) [26], anti-ubiquitin (1:1000, rabbit polyclonal, Cat. Z0458, Dako, CA), anti-GFAP (1:1000, rabbit polyclonal, Cat. Z0334, Dako, CA), anti-Iba1 (1:1000, rabbit polyclonal, Cat. 019–19,741, Wako, VA), and anti-MAP 2A antibody (clone HM-2, 1:1000, mouse monoclonal, Cat. M4403, Millipore/Sigma, MA). To detect primary antibodies, we used biotinylated secondary antibodies and ABC-horseradish peroxidase staining kits (Vector Laboratories, Burlingame, CA). After development by 3,3′-Diaminobenzidine (DAB) (Sigma-Aldrich) substrate and counterstaining with hematoxylin, the slides were cover-slipped and images were taken using an Olympus BX60 microscope or scanned by Aperio® XT System (Leica Biosystems, Buffalo Grove, IL, USA). Blinded observers then reviewed the images and scored the burden of Aβ pathology based on the following criteria; “+++” = heavy Aβ burden with too many deposits to count. “++” = abundant pathology with > 30 deposits per section. “+” = consistent pathology with > 5 deposits per section. “+/−” = inconsistent pathology with < 3 deposits per section.

### Aβ ELISA assay

Sandwich ELISA measurements of Aβ40 and Aβ42 were conducted as previously described [4, 5]. Briefly, the forebrains of the frozen hemi-brains were sequentially extracted in radioimmunoprecipitation assay (RIPA) buffer containing protease inhibitors, followed by 2% sodium dodecyl sulfate (SDS) buffer and 70% formic acid (FA). Aβ levels from brain lysates were evaluated by end-specific monoclonal antibodies (mAbs) anti-Aβ42 (MM26–21.1.3, Aβx-42 specific, Technology #2008–125, T. Golde, Mayo Clinic Ventures) and mAb anti-Aβ40 (MM32–13.1.1, Aβx-40 specific, Technology #2008–120, T. Golde, Mayo Clinic) [26] for capture and horseradish peroxidase–conjugated 4G8 antibody (Covance Inc.) for detection.

### Protein identification by LC-MS/MS

The method used to separate proteins according to their solubility in different detergents has been described previously [42]. Briefly, the method involves sequential extraction and sedimentation in which tissues are first homogenized in PBS and centrifuged at 100,000 x g for 30 min. The supernatant was stored as PBS-S and the pellet fraction was then solubilized in NP40 (0.5%) and centrifuged to produce a pellet that was solubilized in buffers with deoxycholate (2%) and centrifuged. This pellet was then solubilized in buffers with 1% sodium dodecylsulfate (SDS) and centrifuged. The pellets were suspended in Laemmeli buffer [21] and stored as the SDS-P fractions. As in previous work using LC-MS/MS, the primary comparison was between PBS soluble fractions (PBS-S) and SDS insoluble fractions (SDS-P) to identify proteins that are normally readily detectable in soluble fractions but became over-represented in insoluble fractions in mice with Aβ pathology [34, 42]. Twenty microliters of PBS-S fractions or 30 μl of SDS-P fractions, both solubilized in Laemmeli buffer containing β-mercaptoethanol were boiled for 5 min and then separated in 4–20% Criterion™ Tris-HCl precast gels (Bio-Rad;125 V for 15 min in Bio-Rad Criterion gel box), loading every other lane. When the dye front of the sample had migrated ~ 1.5 cm, the electrophoresis was stopped and the gels were stained with Coomassie Blue. The protein smear of each lane was then excised, minced and subjected to in-gel trypsin digestion. The resulting peptides were suspended in 0.1% FA in water for LC-MS/MS. A hybrid quadrupole Orbitrap (Q Exactive Plus) MS system (Thermo Fisher Scientific, Bremen, Germany) was used with high energy collision dissociation (HCD) for MS and MS/MS cycles. An automated Easy-nLC 1000 system (Thermo Fisher Scientific, Bremen, Germany) was interfaced with the MS system in order to conduct a 120 min gradient for chromatography separation. The peptides were separated by a linear gradient from solvent A [0.1% FA (v/v)] to 40% solvent B [0.1% FA (v/v), 80% acetonitrile (v/v)] for 90 min, followed by ramping up to 98% solvent B for additional 30 min.

The tandem mass spectra were extracted using Mascot Distiller (version 2.4), while X! Tandem [The GPM, thegpm.org; version CYCLONE (2010.12.01.01)] was utilized for sample analysis. The mouse UniProt Protein Knowledgebase that had been customized by the addition of human protein data related to expressed transgenes of interest (89,029 entries total) was searched based upon trypsin digestion to identify proteins from peptide spectra. X! Tandem was used to search a reverse concatenated section of the same database. Specifications within X! Tandem and Mascot included a fragment ion mass tolerance of 0.01 Da and parent ion tolerance set to 10.0 PPM. Carbamidomethylated cysteine was input as a fixed modification while variable modifications included glutamine→pyroglutamate at the N-terminus, deaminated glutamate and asparagine, the oxidation of methionine and ubiquitination (Lys-Gly-Gly), as well as ammonia loss on asparagine.

MS/MS-based protein/peptide identifications were confirmed using Scaffold (version Scaffold 4.8.4, Proteome Software Inc., Portland, OR). Subsequent peptide identifications were only established if they surpassed a 50% probability according to the Peptide Prophet algorithm with Scaffold delta-mass correction. X! Tandem yielded peptide probabilities, which were assigned using the Scaffold Local FDR algorithm. The threshold for accepting a particular protein resided at 99% probability and included at least two identified peptides. The Protein Prophet algorithm designated protein probabilities based upon peptide identifications. During instances in which more than one protein was supported by significant peptide evidence, these proteins were arranged into clusters. When a group of proteins could not be distinguished according to MS/MS data, they were grouped according to the principles of parsimony. All mass spectrometry analysis was conducted with the assistance of the University of Florida Interdisciplinary Center for Biotechnology Research Proteomics and Mass Spectrometry Core. The mass spectrometry proteomics data have been deposited to the ProteomeXchange Consortium via the PRIDE [43] partner repository with the dataset identifier PXD017916.

### Statistical and bioinformatics analysis

The unweighted spectrum counts for each protein from the Scaffold data were used for semi-quantification between transgenic and nontransgenic mice (NTg) (numbers of mice by genotype provided in Results). SAINT (significance analysis of interactome) analysis was adopted for probability analysis [7]. SAINTexpress (version 3.6.1) was downloaded from the website (http://saint-apms.sourceforge.net/Main.html) and a computer with Linux operating system was used to conduct the analysis. We combined all 14 NTg mice from different study groups into a single pool of negative controls. This method has more statistical power than pair comparison within the individual study groups because it captures more of the variability in the data from NTg control samples. When doing the analysis, we assumed all the identified SDS-insoluble proteins in a dataset were the preys and the “study group names”, including genotypes and ages, were the “baits”. For example, mouse group “L85_20mo” indicated the genotype is PrP.HuAβ/PS1 and the age is 20 months. The program tested how many proteins were significantly associated with the “bait” L85_20mo, which the statistically determines how many proteins were more prevalent in the SDS-insoluble fraction from the transgenic mice relative to control NTg mice.

In pair-wise comparisons between transgenic and NTg controls, we used the following criteria; the spectral count for a given protein in the SDS-insoluble fractions was ≥5 in every brain sample tested, a G-test assessment of probability for over-representation achieved *p* < 0.05, and the fold change in spectral counts between SDS-insoluble fractions from NTg controls was ≥3 in every transgene positive animal [33, 36]. Additionally, we filtered these data to remove any proteins with a SAINT score less than 0.9, such that the pair-wise comparisons were limited to proteins that passed both statistical methods. Microsoft Excel and SAS JMP® (version Pro 13, Cary, NC, USA) were used to create graphic representations of the data. PANTHER (release 20,190,711) was used to further determine whether the identified proteins were over represented relative to specific biochemical pathways and bioprocesses [28, 29].

## Results

We have previously demonstrated that the accumulation of Aβ in the brains of the PrP.HuAβ/PS1 model is associated with global changes in proteome solubility, such that normally soluble cytosolic proteins become over-represented in SDS-insoluble fractions [42]. Here, we sought to extend this analysis to a panel of APP and APP/PS1 mouse models that deposit human or mouse Aβ peptides, generating either predominantly diffuse or cored-neuritic Aβ deposits [41] (Table 1). These mouse models were generated with transgene vectors that express murine APP, or murine APP with a humanized Aβ sequence, carrying mutations associated with familial AD (Table 1). In some cases, mutant human PS1 was also co-expressed. The expression of transgenes in these mice is driven by vectors with PrP promoters, or by tetracycline-responsive promoters driven in *trans* by the tet-transactivator expressed in vectors driven by the CamKIIα promoter (Table 1).

**Fig. 1:**
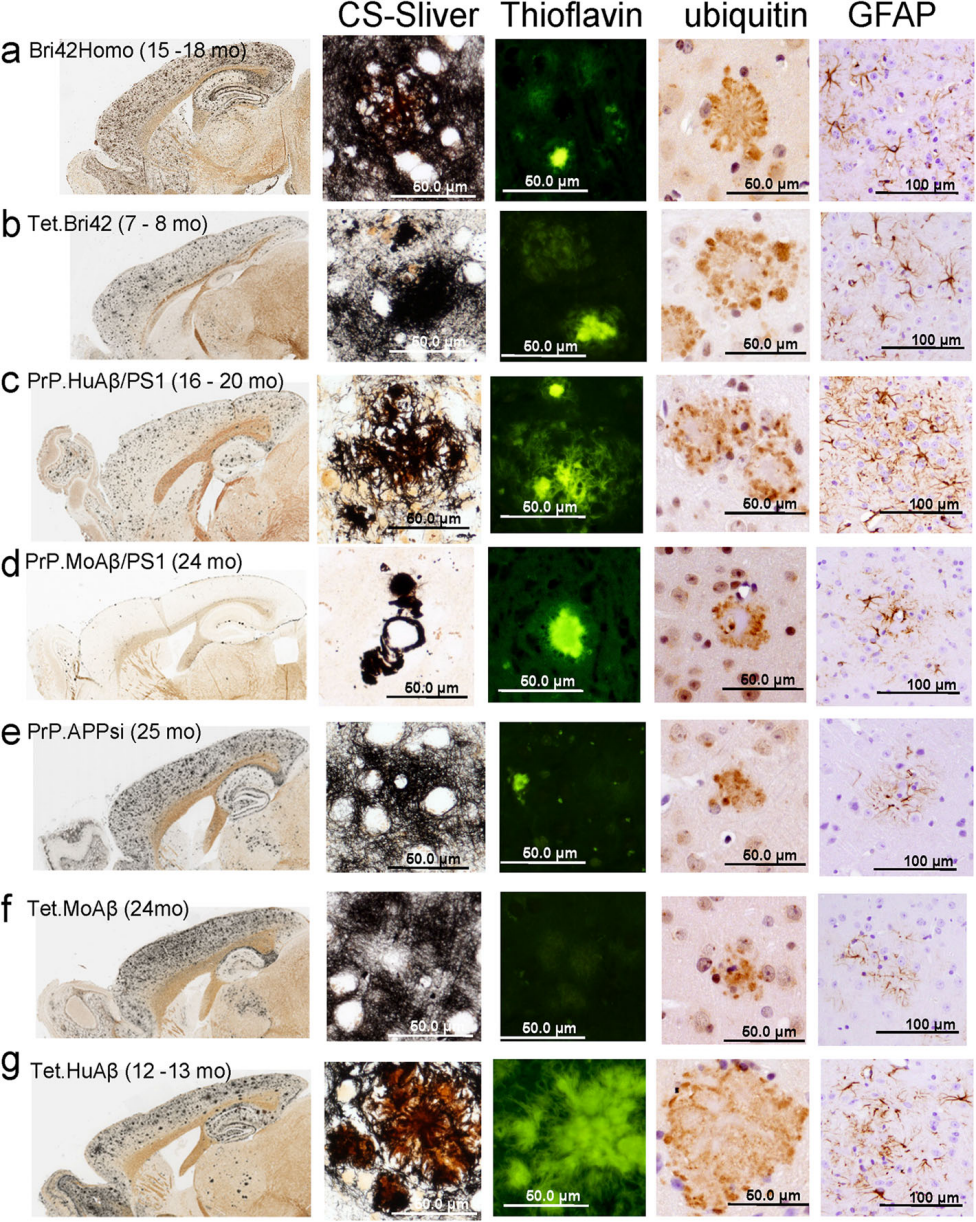
The pathologic profile of the AD-amyloidosis models used in the study. All the images shown here are from mice that provided data for Table 2. The images shown are from tissues stained by the Campbell-Switzer silver (CS-Silver) method to detect all types of Aβ deposition, Thioflavin-S to detect cored deposits, anti-ubiquitin immunostaining to detect neuritic profiles, and anti-GFAP immunostaining to detect astrogliosis. Low power images of the entire hemibrain were captured by scanning microscopy with an Aperio system. At least 3 animals were used for the staining and a representative section from 2 or 3 sections per animal is shown. **a** Bri42Homo, **b** Tet.Bri42, **c** PrP.HuAβ/PS1, **d** PrP.MoAβ/PS1, **e** PrP.APPsi, **f** Tet.MoAβ, **g** Tet.HuAβ. Except for GFAP staining (amplification is 20x, scale bars 100 μm), all the other images were captured at 40x (scale bars 50 μm). See Additional file 1, Fig. S1 a-g for larger images and additional pathologic markers

In addition to the APP and APP/PS1 models, our groups have made mice that develop Aβ amyloidosis by expression of a fusion protein between BRI (ITM2B) and Aβ42, in which the Aβ peptide sequence in fused to the C-terminus of the *BRI* cDNA to generate a protein that is processed in the endoplasmic reticulum to secrete Aβ42 [26]. The original Bri42 mice, generated with the MoPrP. Xho vector, were intercrossed to generate a homozygous line that primarily produces diffuse deposits (Bri42Homo) (Fig. 1a) [19]. Here, we introduce a new line of mice that expresses BriAβ42 using the tet-responsive vector TetPrP. Xho with CamKIIa tTA mice (see Materials and Methods). Similar to the original Bri42 mice [26], the Tet.Bri42 mice exhibited a substantial amount of diffuse Aβ deposition, with cored-neuritic deposits (Table 1; Fig. 1b). These lines of BriAβ42 mice enable us to examine the effects of Aβ42 deposition in the absence of mutant APP or APP/PS1 expression.

For the purposes of this study, we used a simplified nomenclature to identify each mouse line that includes information on the vector used, the species origin of Aβ, and whether mutant PS1 was co-expressed [41] (Table 1). Across the seven different lines of mice that we examine here, we have three lines of mice in which cored-neuritic deposits dominate, three lines of mice in which diffuse deposits dominate, and one line that is mixed (Table 1; Fig. 1a-g).

Because the age at which the different lines first develop Aβ deposits differed, the maximum age that we allowed each model to reach before analysis was varied such that we could compare mice with similar levels of Aβ deposition (Table 2). At the ages selected, the burden of total Aβ deposition as estimated by Campbell-Switzer silver staining was similar across all but one of the different lines of mice (Table 2; Fig. 1a-g). Additionally, at the ages selected, the total burden of Aβ pathology was similar between males and females. Notably, the PrP.MoAβ/PS1 mice develop deposits very late in life and for this line we aged the mice to 24 months, which is near the natural life expectancy of these animals. Even at this advanced age, parenchymal levels of Aβ deposition were relatively low as compared to all other lines of studied here (Table 2, Fig. 1d). At the ages selected, the PrP.HuAβ/PS1 and Tet.HuAβ mice exhibited classic cored deposits with neuritic profiles (Table 2; Fig. 1c and g). The major difference between these lines of mice was a greater level of diffuse Aβ deposition in the Tet.HuAβ mice (Table 1, Fig. 1g). The PrP.MoAβ/PS1 mice exhibited very dense cored deposits that were primarily adjacent to the pia in layer 1 of the cortex or within the corpus callosum (Fig. 1d) [41]. These dense cored deposits were ThioS-positive (Fig. 1d) and Congo Red positive (Additional file 1, Fig. S1 d). Small ThioS and Congo Red positive features were occasionally detected in the brains of the other lines of mice, but they were most abundant in the PrP.HuAβ/PS1 and Tet.HuAβ mice (Table 2; Fig. 1). The Aβ deposits in all lines of mice were recognized by pan Aβ antibody (clone 33.1.1 [6]) and the monoclonal antibody 4G8 [35] (Additional file 1, Fig. S1 a-g). In general, at the ages compared here across these different models, the burden of ubiquitin reactive neuritic deposits, GFAP and Iba1 reactivity appeared to be proportional to the burden of cored, ThioS-positive Aβ deposits (Table 2; Fig. 1a-g, Additional file 1, Fig. S1 a-g).

**Table 2:** Semi-quantitative comparison of the neuropathological features of mouse models at the ages used for LC-MS/MS studies. Scoring criteria described in materials and methods

Mouse models (age assessed)	N	Staining Method							
		Silver	Thio S	Congo Red	33.1.1	4G8	Ubiquitin	GFAP	Iba1
		Diffuse & Cored Aβ	Cored Fibrillary Aβ	Cored Fibrillary Aβ	Aβ	Aβ	Cored-Neuritic Deposits	astrocytes	microglia
**Bri42Homo** **(15–18 mo)**	M3 F1	+++	+	+	++	+	+	++	+
**Tet.Bri42** **(7–8 mo)**	M1 F3	+++	++	++	++	+	+	++	+/−
**PrP.HuAβ/PS1** **(16–20 mo)**	M1 F3	+++	+++	+++	+++	+	++	+++	++
**PrP.MoAβ/PS1** **(24 mo)**	M1 F2	+	+	+	+	+	+/−	+	+/−
**PrP.APPsi** **(25 mo)**	M1 F2	+++	+/−	+/−	+++	+/−	+/−	+	+/−
**Tet.MoAβ** **(24 mo)**	M1 F2	+++	+/−	+/−	+++	+/−	+/−	+	+
**Tet.HuAβ** **(12 mo)**	M2 F2	+++	+++	+++	+++	++	+++	+++	++

### Changes in proteome solubility in Aβ mouse models

To identify cytosolic proteins that become over-represented in SDS-insoluble fractions in these different models of AD type amyloidosis, we followed the same methods of sequential detergent extraction that we have used in prior studies, using LC-MS/MS with label-free spectra counting methods [34, 42]. One criteria for identification as a soluble cytosolic protein was that the protein be readily detected in PBS soluble fractions. As detailed in Materials and Methods, we used two statistical approaches to analyze the spectral count data and identify over-represented proteins in SDS-insoluble fractions. In one type of analysis, we used pair-wise comparisons of the SDS-insoluble peptide spectral counts for each identified protein in transgenic mice relative to those of NTg control animals within its own genetic background and age range (Table 3). The Bri42 homozygous mice were generated by inbreeding between homozygous littermates that were originally generated on the B6/C3H hybrid background, and thus data from NTg B6/C3H mice were used as the controls. The pair-wise analysis we used set rather stringent criteria for a positive detection of a protein as over-represented in SDS-insoluble fractions. We set the threshold at a minimum detection of 5 spectra for a given protein in each sample from transgenic animals with a minimum of a 3-fold change in spectral counts between controls and transgenics. The pair-wise analysis incorporated a G-test statistical algorithm to determine which proteins were statistically over-represented in SDS-insoluble fractions. To complement the pair-wise analysis, we also used SAINT (Significance Analysis of INTeractome) analysis [7] in a manner similar to what we recently described [34]. The SAINT analysis provides an alternative statistical method to determine the probability that spectra for a given protein are over-represented in the SDS-insoluble fraction. When using SAINT analysis to identify over-represented proteins, we elected to group all 14 data sets from NTg as a single control group for the transgenic data sets. To assess whether there might be age-associated changes in proteome solubility in the NTg, we conducted a SAINT analysis comparing data from five 16–24 month old animals to two 2.5 month old animals. A short list of proteins were detected as over-represented in the insoluble fractions from the older mice (0.9 confidence score in SAINT), including Tubb3, Tubb6, Mbp, Uba52, Rsp27a and Hist1h1e (Additional file 2, Table S1). These data indicate that age was a minimal factor in alterations in proteome solubility, and that combining all NTg data sets as a single control group would not significantly increase the false discovery rate.

**Table 3:** Summary of data on cytosolic proteome solubility

Mouse model	Strain background	Age assessed	Total Aβ burden assessed by CS-silver stain	# Animals	# of cytosolic proteins identified as over-represented in SDS insoluble fractions		
					Pairwise only	Pairwise and SAINT > 0.9	SAINT Score only
**PrP.HuAβ/PS1**	B6/C3	20 mo	+++	3	101	87	103
**PrP.MoAβ/PS1**	B6/C3	24 mo	+	2	2	0	0
**Bri42Homo**	B6/C3	15 mo	+++	3	9	0	5
**PrP.APPsi**	B6/C3	25 mo	+++	3	3	0	4
**NTg**	B6/C3	12 mo	–	1			
		16 mo	–	1			
		20 mo	–	2			
**Tet.HuAβ**	B6/FVB F1	2.5 mo	–	3	0	0	0
**Tet.HuAβ**	B6/FVB F1	13 mo	+++	4	89	80	139
**Tet.MoAβ**	B6/FVB F1	24 mo	+++	3	131	38	48
**Tet.Bri42**	B6/FVB F1	7–8 mo	+++	4	32	12	15
**NTg**	B6/FVB F1	2.5 mo	-	2			
		13 mo	-	6^a^			
		24 mo	-	2			

^a^Includes 4 mice that were treated with Dox for 1 or 4 weeks (2 each). The raw data used to produce this table are available as excel files in Additional files 3, 4 and 5, Tables S2, S3 and S4

Using a stringent criteria in which proteins that met significance by both pairwise comparison (strain background and age-matched) and SAINT score comparison (> 0.9 probability), we identified proteins in each amyloidosis model that were over-represented in SDS-insoluble fractions (Table 3). We observed that the PrP.HuAβ/PS1 and Tet.HuAβ mice, which primarily develop cored-neuritic deposits of Aβ, had the highest number of cytosolic proteins that met our stringent criteria for over-representation in SDS-insoluble fractions (87 and 80 proteins, respectively). As expected from prior studies [42], the number of proteins identified as over-represented in SDS-insoluble fractions from the forebrains of PrP.HuAβ/PS1 mice was lower in younger 9–12 month old animals, which have low burdens of Aβ deposition, indicating age-progressive proteostatic disruption (Additional file 3, Table S2).

**Fig. 2:**
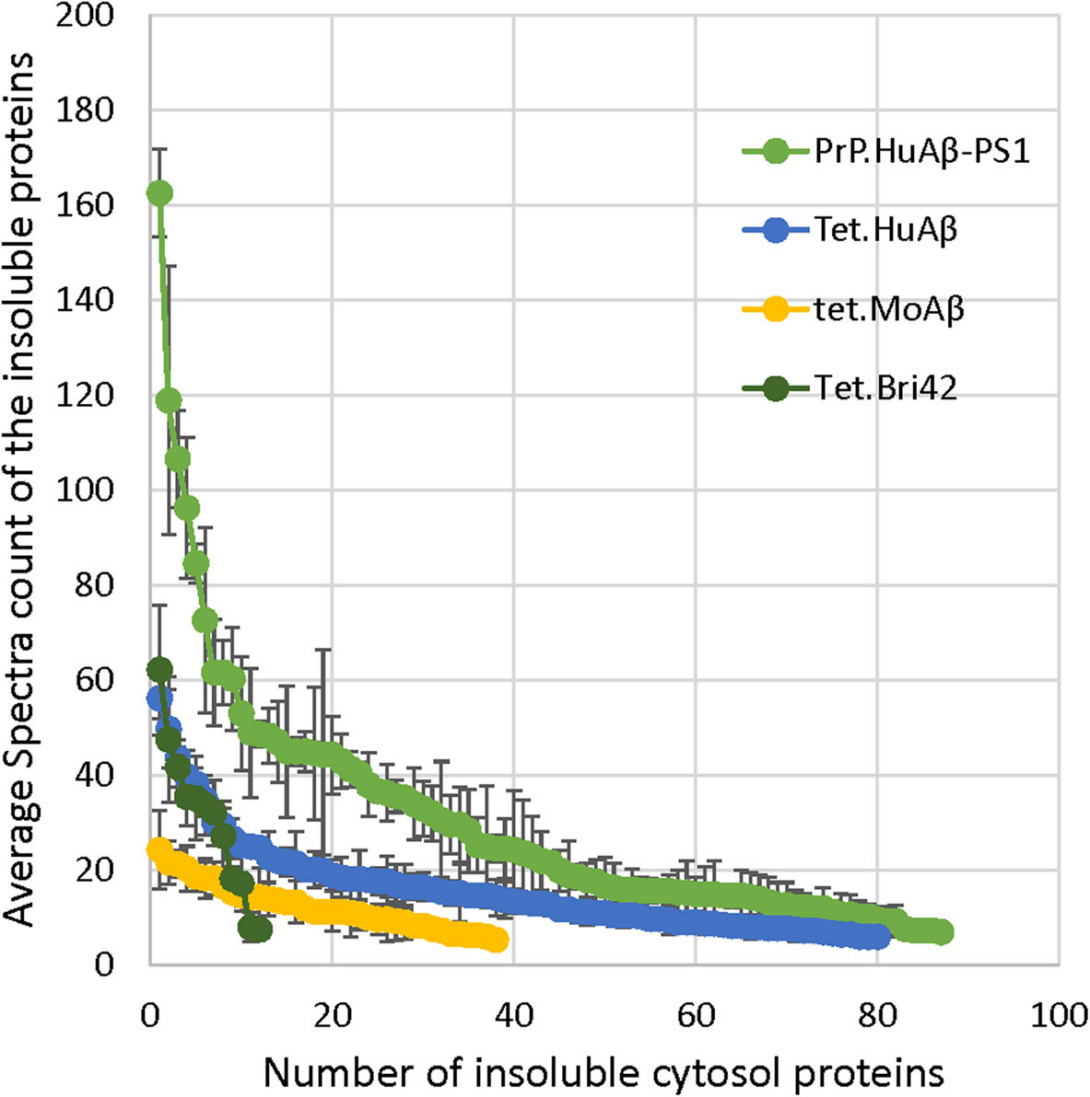
Analysis of changes in proteome solubility across the different models of amyloidosis. This graph plots the proteins identified by pair-wise comparison combined with SAINT analysis (≥ 0.9 probability and spectral count ≥5) for each protein identified in the SDS-insoluble fraction from each line of transgenic animals (raw data in Additional files 3 and 4, Tables S2 and S3). The X- axis was generated by the rank ordering of proteins that met criteria as over-represented in SDS-insoluble fractions from the highest to lowest spectral count numbers with the Y-axis plotting the average number of spectral counts (error bars indicate standard deviation). Note that the rank order of proteins identified differed between lines and therefore the data points between different lines of mice should not be viewed as aligning. Rather, the graphs provide an indication of relative number of spectra counts for proteins identified within each line

The spectral counting method we used provides a semi-quantitative measure of the abundance of any given protein in the SDS-insoluble fractions, indicating of the degree of change in proteome solubility. To compare the data between different mouse lines of amyloidosis, we graphed the spectral counts for a given protein against its rank order of abundance for each of the lines (Fig. 2). It is important to note here that the rank order of protein abundance between lines of mice were different and therefore, the data points are not necessarily aligned for a given protein. The graph provides a visual representation of the variation in the total number of proteins that met our stringent criteria (pair-wise and SAINT combined) for over-representation as well as the relative number of spectra for each of the identified proteins. In the brains of 20 month old PrP.HuAβ/PS1 mice there were 40 proteins in which the average spectral counts in SDS-insoluble fractions were 20 or higher (Fig. 2). The cytosolic proteins with the highest number of spectra included Gapdh, DnmI, Ckb, Hspa8, Eno1, Stxbp1, Aldoa, Ywhaz, Uba1, Hsp90aa1, and Ywhab (fold increases relative to age-matched NTg controls were 5x, 5x, 8x, 4x, 9x, 7x, 9x, 7x, 13x, 10x, and 8x). For the vast majority of proteins in which the average spectral counts in the SDS-insoluble fractions were between 20 and 50, the average number of spectral counts for the same protein in SDS fractions from NTg mice ranged between 0 and 3 (Additional file 3, Table S2). Although the total number of proteins that were identified as over-represented in the SDS-insoluble fraction of the 13 month old Tet.HuAβ mice was similar to the number in 20 month old PrP.HuAβ/PS1 mice, the number of spectra for each identified protein was lower (Fig. 2). Similarly, in the 24 month old Tet.MoAβ mice, the number of spectra per protein identified was lower (Fig. 2). Intriguingly, although the number of proteins that met statistical significance in the Tet.Bri42 mice was relatively low (12 proteins), the average spectral counts for these tended to be relatively high. In the 25 month old PrP.APPsi mice, the 15 month old Bri42Homo mice, and the 24 month old PrP.MoAβ/PS1 mice, the number of cytosolic proteins that achieved significance by pairwise comparison combined with SAINT scoring was zero (Additional files 3 and 4, Tables S2 and S3). Collectively, these data indicate that as compared to mice with mixed pathology or predominantly diffuse Aβ deposits, the PrP.HuAβ/PS1 and Tet.HuAβ models, which have high levels of cored-neuritic Aβ deposits, have more severe changes in proteome solubility.

We identified the 40 most abundant proteins in SDS-insoluble fractions of brains from Tet.HuAβ and PrP.HuAβ/PS1 mice (Additional file 6, Tables S5 and S6, respectively) and determined that 23 of these proteins that were found in both lines of mice. Within the entire dataset for proteins over-represented in SDS-insoluble fractions for these two models, there were 50 proteins common between the models with 71 unique to one model or the other (Additional file 1, Fig. S2.). Using the PANTHER Overrepresentation Test to analyze the insoluble cytosolic proteins, we found that in both Tet.HuAβ and PrP.HuAβ/PS1 mouse models, the class of proteins that most commonly showed altered solubility included chaperone proteins (*p* values =5.17 × 10^− 11^, 3.56 × 10^− 16^, respectively), while pathway-analysis indicated glycolysis enzymes were over-represented (p value =1.04 × 10^− 7^, 1.68 × 10^− 18^, respectively). According to biological-process-analysis, proteins involved in neurotransmitter secretion were the most significant (p value = 8.40 × 10^− 6^) in the Tet.HuAβ mice and proteins involved in carbohydrate metabolic processes were most significant (p value = 2.23 × 10^− 11^) in the PrP.HuAβ/PS1 mice. These data indicate that there were both shared and unique signatures of proteome insolubility between these models.

In the current study, we have replicated our prior analysis of proteome solubility in the PrP.HuAβ/PS1 mice [42]. In the prior study of 16 mo old mice, 28 proteins satisfied our criteria for over-representation (at least 5 spectra were found in the transgene positive samples and the number of spectra were at least 5-fold higher in the transgene positive samples compared to NTg controls). In our current study, among the 40 proteins that showed the greatest differential in peptide spectral counts between controls and transgenics, there were 20 proteins that were identical to the 28 from the first study (Additional file 6, Table S5 – column [a]). In our previous study, we used a QSTAR® XL instrument (Applied Biosystems, Foster City, CA, USA), which is a hybrid quadrupole time-of-flight mass spectrometer. In the present study, we used the Q Exactive Plus MS system (Thermo Fisher Scientific, Bremen, Germany). A comparison of the distribution of spectral count numbers between two studies showed that the new study has a much higher number of spectral counts for all proteins identified with twice as many proteins identified (Additional file 1, Fig. S3). Notably, in the current study we have adapted a statistical algorithm for analyzing protein interaction data for use in our paradigm [34]. The current list was supported by the SAINT score algorithm, which factors protein molecular weight in its calculation of statistical probability of over-representation. Many of the proteins that met criteria for statistical significance in the current study of PrP.HuAβ/PS1 mice were also detected in the prior study, but failed to meet our original criteria for statistical significance (see Additional file 6, Table S5, footnote b). Thus, we conclude that the present study of the PrP.HuAβ/PS1 mice provides an indication of the reproducibility of our approach.

### Relationships between changes in protein solubility and levels of Aβ peptide spectra in SDS-insoluble fractions

**Fig. 3:**
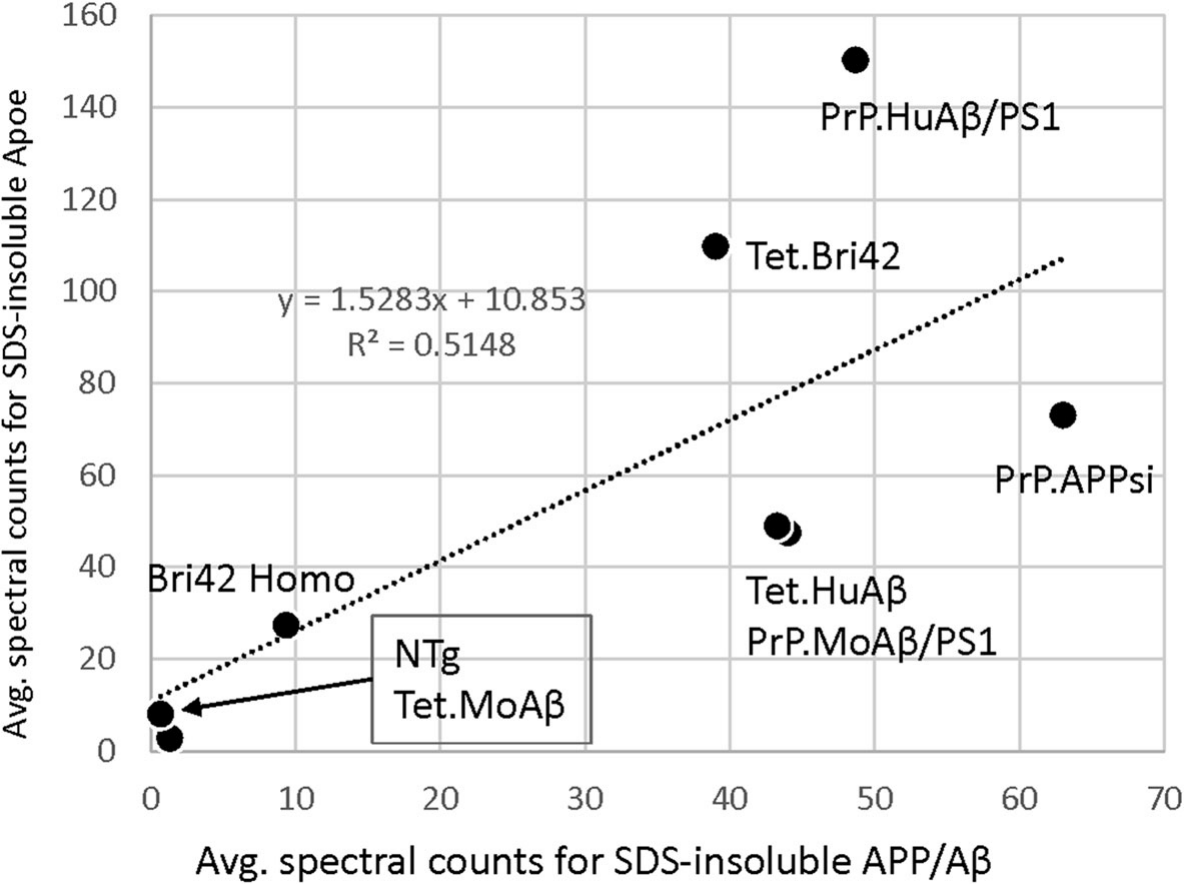
Correlation between spectral counts for APP/Aβ and Apoe in SDS-insoluble fractions. The average of spectral counts of Aβ and ApoE in SDS-insoluble fractions from the brains of mice of each genotype (Table 4) were used for the regression analysis (*R*^*2*^ = 0.51, *p* < 0.05)

In analyzing our spectral count data, it was clear that the abundance of Aβ in the SDS-insoluble fractions of brains from these amyloidosis models was not predictive of how many cytosolic proteins were identified as over-represented in insoluble fractions. The average number of spectral counts for Aβ in SDS-insoluble fractions was similar in the Tet.Bri42, PrP.MoAβ/PS1, PrP.HuAβ/PS1, Tet.HuAβ, Tet.Bri42, and PrP.APPsi models whereas the number of cytosolic proteins identified as over-represented in insoluble fractions ranged from 0 to 87 (Table 4). By contrast, Apoe is a secreted protein that is known to be associated with Aβ deposits [23, 31] and, in mice that exhibit some level of cored-neuritic pathology, the spectral counts for Apoe tended to be proportional to Aβ spectral counts (Table 4; Fig. 3). In tandem with Apoe, immunoreactivity for the secreted protein Clusterin (Clu) [25], or Apoj, has also been described as associated with cored-neuritic deposits; concordantly, the spectral counts for Clu were highest in in the SDS-insoluble fractions of mice exhibiting cored-neuritc deposits (Table 4).

**Table 4:** Summary of data for APP/Aβ, Apoe, and Clusterin spectral counts in SDS-insoluble fractions

Mouse Line	PrP. MoAβ/PS1	PrP. HuAβ/PS1	Tet. HuAβ	Tet. Bri42	Bri42 Homo	PrP. APPsi	Tet. MoAβ
**Amyloid pathology type**	C	C > > D	C > D	D ≈ C	D > C	D > > C	D
**Age (mo)**	24	20	13	8	15	25	24
**Number of LC/MS-MS**	2	3	4	4	3	3	3
**Total # over-represented cytosolic proteins in SDS-insoluble fractions by pairwise comparison plus SAINT > 0.9**	0	87	80	12	0	0	38
**Avg. # SDS-insoluble Aβ spectra**	44	49	43	39	9	63	1
**Avg. # SDS-insoluble Apoe spectra**	48	150	49	110	27	73	8
**Avg. # SDS-insoluble Clu spectra**	6	33	23	16	5	10	4

**Fig. 4:**
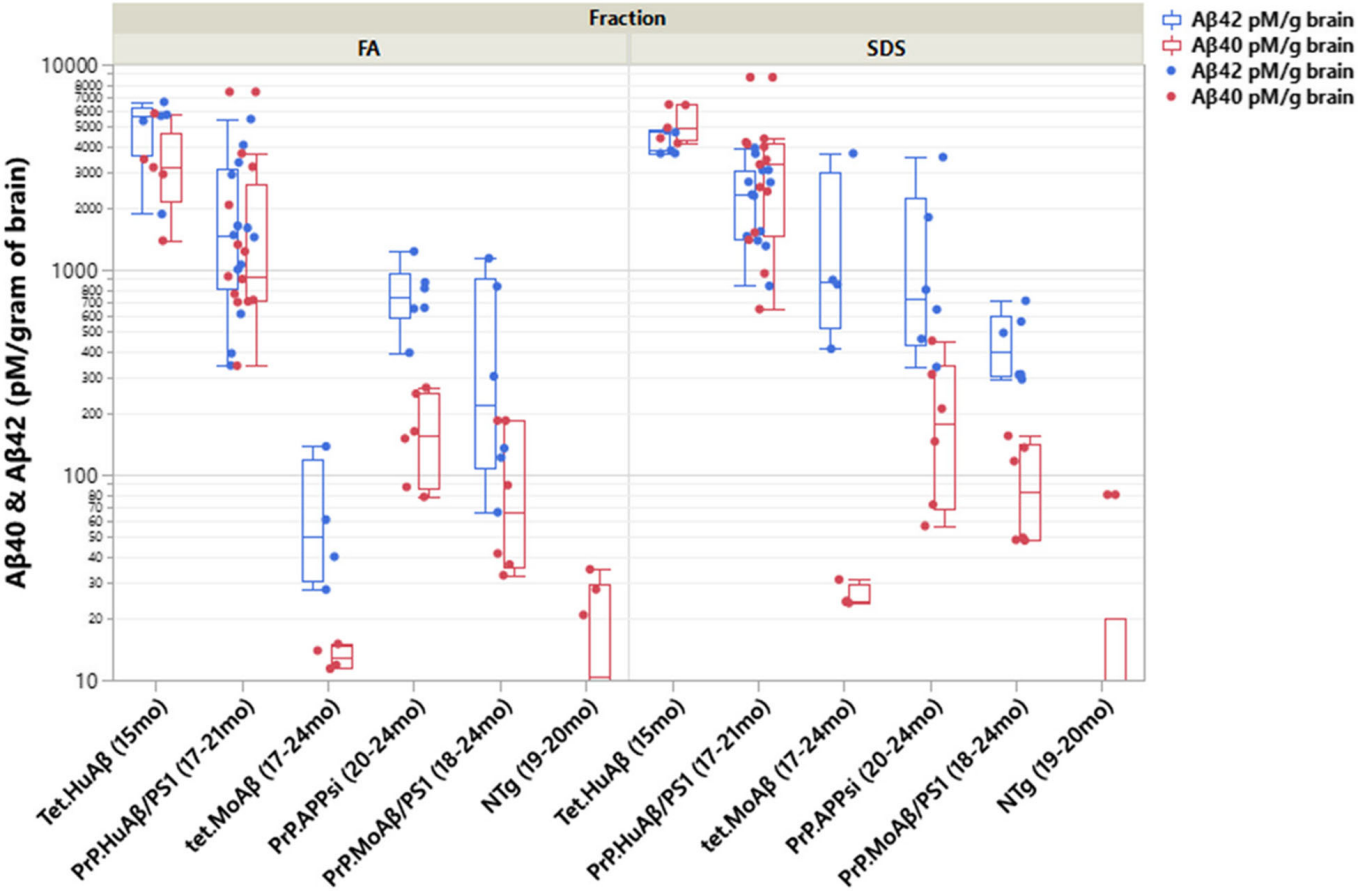
Aβ 40 and 42 levels in the APP and APP/PS1 amyloidosis models used in this study. The levels of Aβ in the forebrains of mice were measured by ELISA assay as described in Materials and Methods. The ages of the mice at the time of analysis is noted in the x-axis of the figure. The blue box plots and dots mark the levels of Aβ42 whereas the red dots and box plots mark the levels of Aβ 40. Each dot represents a value detected from one animal. The average value in each set of samples is noted by the horizontal lines in the box plots. The number of animals harvested for each genotype were as follows: Tet.HuAβ (*n* = 5), PrP.HuAβ/PS1 (*n* = 12), Tet.MoAβ (*n* = 4), PrP.APPsi (*n* = 6), and PrP.MoAβ/PS1 (*n* = 6). Since the Bri42 and Tet.Bri42 mice do not produce Aβ40, they were not included in this study, which was meant to assess the relative levels of Aβ40 and 42 across the APP and APP/PS1 models that could produce both peptides

The amyloidosis models generated by expressing humanized APP produce mixtures of both Aβ40 and Aβ42 and here we compare the composition of the deposited Aβ in these models side by side with sandwich ELISA assays (Fig. 4). The brains of Tet.HuAβ and PrP.HuAβ/PS1 mice, which develop primarily cored deposits, accumulate high levels of FA-soluble and SDS-soluble Aβ40 and Aβ42. The brains of Tet.MoAβ mice, which develop diffuse Aβ deposits, had relatively low levels of FA-soluble Aβ42 and high levels of SDS-soluble Aβ42. As previously observed [41], the brains of these mice had low levels of Aβ40 in these fractions. In the PrP.APPsi mice, which at the ages analyzed exhibited both diffuse and cored deposits, the levels of FA- and SDS-soluble Aβ42 were similar with relatively low levels of Aβ40. Notably, although the overall burden of deposits in the brain parenchyma of the PrP.MoAβ/PS1 mice was relatively low even at advanced ages, this line of mice shows significant deposition in the pia which accounts for most of the Aβ42 detected by ELISA (Fig. 4, see Additional file 1, Fig. S1 d for a larger image). Across all the models based on expression of APP transgenes, the PrP.HuAβ/PS1 and Tet.HuAβ mice were unique with a high frequency of cored-neuritic deposits and roughly equivalent levels of FA- and SDS-insoluble Aβ40 and Aβ42.

### Partial mitigation in proteome solubility by suppressing mutant APP transgene expression

**Fig. 5:**
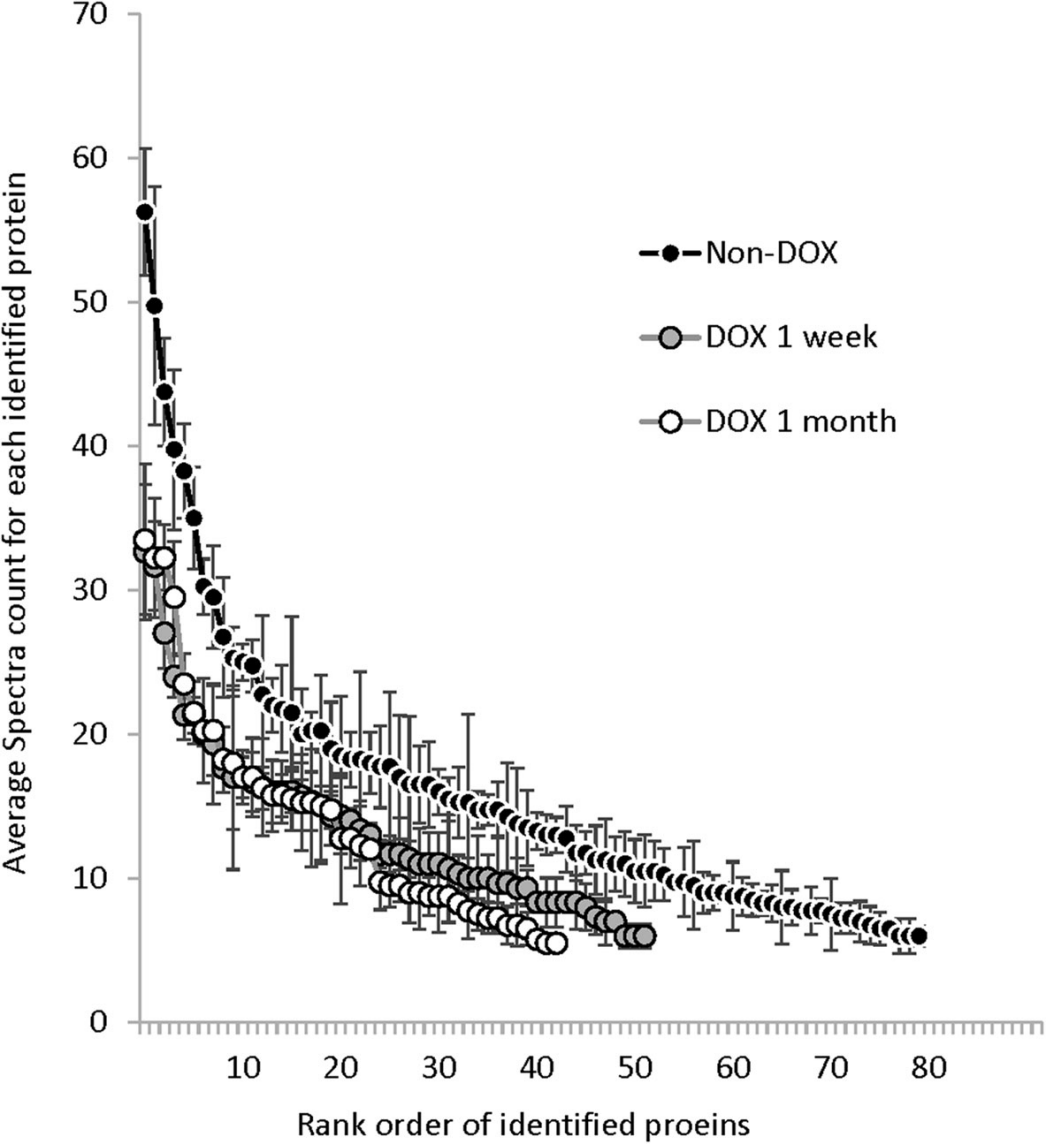
Analysis of SDS-insoluble proteins in Tet.HuAβ mice before and after DOX treatment. The graph design follows that of Fig. 2. The X- axis was generated by the rank ordering of proteins that met criteria as over-represented in SDS-insoluble fractions from mice that were untreated or treated with Dox for 1 or 4 weeks (raw data in Additional file 4, Table S3). The rank order for each data set is from the highest to lowest spectral count numbers with the Y-axis plotting the average number of spectral counts (error bars indicate standard deviation). Note that the rank order of proteins identified differed between the treatment groups and therefore the data points between different lines of mice should not be viewed as aligning

The expression of the APP transgene in Tet.HuAβ mice can be suppressed when mice are fed diets containing Dox [14]. In this model, suppressing transgene expression in 13-month-old mice for 1 week can produce partial improvements in cognitive behavior despite little or no change in the burden of Aβ deposits [27]. Following the same paradigm, we treated 12–13 month-old Tet.HuAβ mice with Dox for 1 week or 1 month and then harvested forebrains for LC-MS/MS analysis of the insoluble proteins. We found that the total number of proteins that met our stringent criteria for over-representation in the brains of the Dox treated animals was lower at 1 week with modest further reduction after 1 month on Dox (Table 5). When the data was analyzed by rank order, we observed that the most abundant proteins in the Dox treated mice showed lower spectral counts than the most abundant proteins in the untreated mice (Fig. 5). By contrast, the average number of Aβ spectra in SDS insoluble fractions in the brains of mice treated with Dox were not statistically different from that of untreated mice (Additional file 4, Table S3). Overall, our analysis indicated that changes in proteome solubility were partially mitigated by suppressing APP expression, an outcome that suggests that the persistent Aβ deposits are at least partially responsible for causing the changes in proteome solubility that was detected in the Tet.HuAβ mice.

**Table 5:** Summary of data for insoluble cytosolic proteins in 13 month old Tet.HuAβ treated with Dox

Genotypes	Age assessed	Total Aβ burden assessed by CS-silver stain	# Animals	# of cytosolic proteins identified as over-represented in SDS insoluble fractions
				Pair-wise analysis Combined with SAINT > 0.9
**Tet.HuAβ**	2.5 mo	–	3	0
	13 mo	+++	4	80
	13 mo + Dox 1wk	+++	3	52
	13 mo + Dox 4 wk	+++	4	43
**NTg**	2.5 mo	-	2	NA
	13 mo	-	2	
	13 mo + Dox 1wk	-	2	
	13 mo + Dox 4 wk		2

### Comparative analysis of SDS-insoluble proteome across different amyloidosis models

**Fig. 6:**
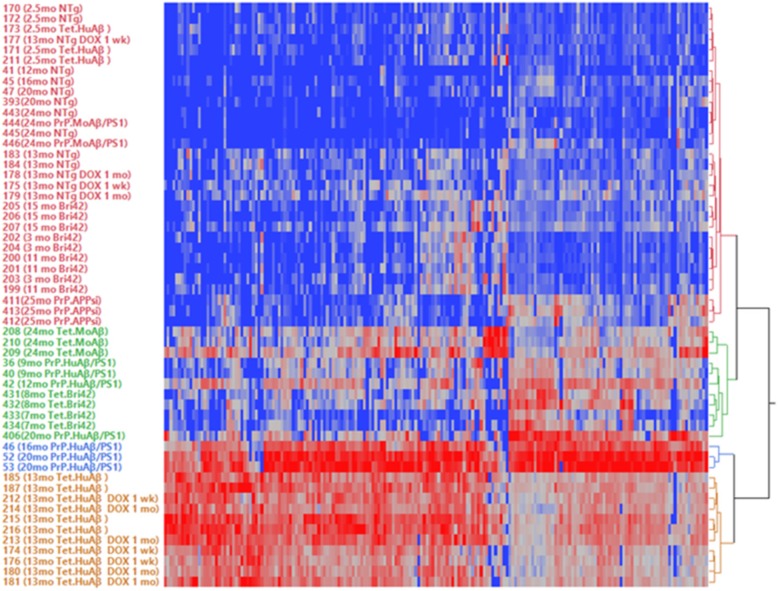
Two-way clustering of the data sets for SDS-insoluble cytosolic proteins identified by LC-MS/MS from all groups of the mice used in the study. Cluster analysis was performed on the SDS-insoluble peptide spectra data for 191 total proteins identified across all the mice, using the SAINT scoring criteria (Additional file 5, Table S4). These 191 proteins were selected by outer join merging of all the SDS-insoluble proteins from each group. Red is indicative of the highest number of peptide spectra for a given protein whereas blue indicates the lowest number of spectral count for a given protein. The figure was generated by JMP Pro Statistical Discovery from SAS. Note that the number in front of each animal identification represent the run number that produced the LC-MS/MS data. Although there were some small influences of batch in the data clustering, the three main clusters consisted of data sets that were run at very different times in the process

We used cluster analysis to identify how the proteome datasets from the different mouse models of amyloidosis are related to each other (Fig. 6). We found that the datasets from the 13 mo old Tet.HuAβ mice, whether treated with Dox or not, cluster together as a distinct group relative to all other datasets (Fig. 6). Whereas two of the 20 mo old and the 16 mo old PrP.HuAβ/PS1 mice were related to the Tet.HuAβ mice, one of the 20 mo old animals clustered into a second group of transgenics that included older Tet.MoAβ and Tet.Bri42 mice, and younger PrP.HuAβ/PS1 mice. The largest cluster was made up of NTg mice (all ages), old PrP.MoAβ/PS1 mice, old PrP.APPsi mice, all Bri42Homo mice regardless the age, and young Tet.HuAβ mice, showing that the insoluble proteome from all of these lines were very similar to NTg mice. Collectively, these data demonstrate that the insoluble proteomes of these different models of Alzheimer-type amyloidosis exhibit very different profiles of changes in protein solubility that are partially driven by total Aβ burden, the neuropathological features of Aβ deposits, and the expression pattern of the amyloidogenic transgenes.

## Discussion

In the present study, we provide a comparison of seven different mouse models that develop Aβ deposits composed of human or mouse Aβ peptides. These models, which were developed with different transgene constructs, produce a range of neuropathologic features characterized by divergent ages of onset of Aβ deposition, composition of Aβ peptides within the deposits, and morphology of the deposits. Using altered solubility of cytosolic proteins as a general biomarker of proteostasis dysfunction, we observe that cored, Thio-S positive, neuritic deposits that are composed of both Aβ40 and Aβ42 cause a greater degree of proteostasis dysfunction in the brains of mice than other types of Aβ pathology.

It has been widely recognized that different types of Aβ pathology consist of biochemically distinct forms of Aβ [18]. The Aβ peptides in cored, ThioS-positive deposits are generally insoluble in detergent (SDS) and require FA or guanidine denaturation to be solubilized. The Aβ peptides associated with diffuse deposits are generally soluble in SDS and are ThioS-negative. Cross-sectional neuropathological studies of aged humans seem to indicate that diffuse Aβ deposits develop first with cored deposits becoming more prevalent with age and in higher abundance in cognitively impaired individuals, suggesting a progressive relationship between the two types of pathology [3]. Notably in the PrP.APPsi mice, we observe both diffuse and cored Aβ pathology, but ultimately diffuse deposits predominate. By contrast, cored-neuritic deposits predominate in the Tet.HuAβ model, which express essentially the same APP cDNA transgene as the PrP.APPsi mice. These observations suggest that different types of Aβ pathology can develop and evolve independently.

Across our panel of models, there are variations in the strain background, the promoter elements used in the transgene vector constructs, the mutations within APP that promote Aβ secretion, and whether mutant PS1 is co-expressed with APP [16]. These variables appear to give rise to the diversity in neuropathologic features we observe. It is relatively easy to see how variations in the sequence of the Aβ peptides themselves, such as species variations used here, could influence the properties of misfolded Aβ within a model. When two different models that should produce Aβ peptides of exactly the same sequence produce morphologically distinct Aβ pathologies, such as the PrP.MoAβ/PS1 and Tet.MoAβ models, then we can assume that other factors must be contributing to generating the distinct pathologic morphologies. Moreover, the two models that utilize PrP vectors to express APPswe with mutant PS1 share similarities in terms of preponderance of cored deposits despite the fact that the Aβ peptides are of different species (PrP.HuAβ/PS1 compared to PrP.MoAβ/PS1). By contrast, the Tet.MoAβ and Tet.HuAβ mice use the same constructs to drive transgene expression and yet show very different pathologic morphologies. Collectively, the data suggest that the ultimate morphology of the Aβ deposits is dictated by some aspect of the cellular origin of the Aβ, modulated by Aβ primary sequence, with some influence of mutant PS1 on the processing of mutant APP to produce different mixtures of Aβ peptides.

In order to analyze changes in proteome solubility in tissues from animals with similar amyloid burdens, it was necessary to use animals of different ages; however, across our groups of animals it is possible to compare models of similar age and amyloid burden. For example, the cohort of Tet.HuAβ mice (13 mo of age) is comparable to the cohorts of Tet.Bri42 (8 mo of age) and Bri42Homo mice (15 mo of age). Despite similar levels of total Aβ burden, the Tet.HuAβ mice had far higher numbers of proteins that aberrantly fractionated to SDS-insoluble fractions, with relatively higher spectral counts for the proteins identified. The Tet.HuAβ mice develop large cored-neuritic deposits that robustly bound ThioS and Congo Red. These mice accumulate high levels of both Aβ40 and Aβ42 in both the SDS and FA fractions. By contrast, evidence for changes in cytosolic protein solubility were very limited in the Tet.Bri42 and Bri42Homo mice, which deposit only Aβ42 and produce mixed pathology that includes diffuse as well as cored-neuritic deposits. Similarly, we can compare the PrP.HuAβ/PS1 mice to the PrP.APPsi and Tet.MoAβ models (all aged to 20–24 months). The Aβ pathology of the PrP.HuAβ/PS1 mice is very similar to the Tet.HuAβ mice and likewise the number of proteins that we observed to lose solubility was relatively high. In the PrP.APPsi mice, which show mixed pathology of diffuse and cored deposits composed primarily of Aβ42, no cytosolic proteins were observed to lose solubility. By contrast, the number of proteins showing aberrant insolubility in SDS in the Tet.MoAβ mice, which show only diffuse deposits, was higher than that of the Tet.Bri42 mice of a similar age. At present we cannot fully explain why models such as the Tet.BriAβ42 or Bri42Homo mice show fewer changes in proteome solubility despite high burdens of Aβ42 deposition. Overall, the pattern that emerges is that increased levels of cytosolic protein insolubility is associated with the deposition of both Aβ40 and 42 into cored-neuritic deposits.

Our study of Tet.HuAβ mice treated with Dox for varied intervals revealed that, within 1 week of suppressing mutant APP expression, there was a reduction in the levels of insoluble cytosolic proteins that changed only modestly with 3 additional weeks of Dox treatment. Over these short treatment intervals, there was no significant change in the number or appearance of Aβ deposits in this model, consistent with earlier reports [14, 27]. This finding suggests that the changes in protein solubility may be partially mediated by a factor other than the persistent Aβ deposits. Whether this factor is over-expressed APP or some proteolytic derivative such as sAPPα or CTFβ, or a labile pool of Aβ that clears quickly after APP expression is suppressed, remains to be determined. It is also possible that Dox treatment had some direct effect on proteome solubility as Dox has been shown to modulate the aggregation of systemic amyloids such as transthyretin [1]. Importantly, the persistent neuritic deposits in this model are associated with sustained changes in proteome solubility, implicating these structures as key mediators of altered proteome solubility.

## Conclusions

The present study reveals an underappreciated complexity in the types of Aβ pathology exhibited by different lines of transgenic mice that appears to be dictated by aspects of the transgene constructs expressed, and whether mutant presenilin is co-expressed with APP. Diffuse Aβ deposits appear to be much less disruptive to cellular protein solubility than cored-neuritic deposits. Mice that develop Aβ deposits that are primarily composed of misfolded Aβ42 showed surprisingly modest levels of proteome insolubility whereas the presence or both Aβ40 and Aβ42 in cored-neuritic structures was associated with more robust changes in cytosolic protein solubility. Collectively, our findings illuminate the biological diversity of CNS responses to different types of Aβ pathology and provide evidence that cored-neuritic deposits of Aβ40/Aβ42 are associated with a greater disruption of CNS proteostasis function.

## Supplementary information

**Additional file 1: Figure S1.** The pathologic profile of the AD-amyloidosis models used in the study. All the images shown here are from mice that provided data for Table 2. The images shown are from tissues stained by the Campbell-Switzer silver (CS-Silver) method to detect all types of Aβ deposition, anti-Aβ antibodies 33.1.1, and 4G8 to detect Aβ, Thioflavin-S and Congo Red to detect cored deposits, anti-ubiquitin immunostaining to detect neuritic profiles, anti-GFAP immunostaining to detect astrogliosis, anti-Iba1 to identify microglia, and anti-Map 2 to show the distruption of neuropil by Aβ deposits. Images of Silver stains, Thioflavin-S, anti-ubiquitin, and GFAP are reproductions of images shown in Fig. 1 with digital enlargement. Low power images of the entire hemibrain were captured by scanning microscopy with an Aperio system. At least 3 animals were used for the staining and a representative section from 2 or 3 sections per animal is shown. a) Bri42Homo, b) Tet.Bri42, c) PrP.HuAβ/PS1, d) PrP.MoAβ/PS1, e) PrP.APPsi, f) Tet.MoAβ, g) Tet.HuAβ. Except for GFAP staining (amplification is 20x, scale bars 100 μm), all the other images were captured at 40x (scale bars 50 μm). Representative scale bars are provided. **Figure S2.** Venn diagram of the overlap of Tet.HuAβ and PrP.HuAβ/PS1 mice analyzed based upon LC-MS/MS analysis of SDS-insoluble proteins. These diagrams are based on the pair-wise statistical analysis combined with the SAINT scoring method of identifying proteins that were over-represented SDS-insoluble fractions from the brains of the Tet.HuAβ mice versus PrP.HuAβ/PS1 mice. **Figure S3.** Comparison of mass spectral data between instruments. The graph shows the relationship between spectral count data and protein identification data across two datasets generated with different instruments. The blue line graphs data obtained with a QStar instrument used in a study published in 2013 [42]. The red line graphs data obtained for the current study with a QE+ instrument. In the data for the current study, more total proteins were identified and the spectral counts for these proteins were higher than what was observed in the earlier study.

**Additional file 2: Table S1.** Excel spreadsheets for SAINT analysis of spectral count data for the SDS-insoluble proteins identified from young vs old NTg mice.

**Additional file 3: Table S2.** Excel spreadsheets that list the identity and spectral count data for the SDS-insoluble proteins identified from each model that have the B6/C3 background (See Table 3) using pair-wise analysis combined with SAINT scoring. Each model is tabulated independently. These mice include NTg, PrP.HuAβ/PS1, PrP.MoAβ/PS1 and Bri42 Homo mice.

**Additional file 4: Table S3.** Excel spreadsheets that list the identity and spectral count data for the SDS-insoluble proteins identified from each model that have the B6/FVB F1 background (See Table 3) using pair-wise analysis combined with SAINT scoring. Each model is tabulated independently. These mice include NTg, Tet.HuAβ, Tet.MoAβ and Tet.Bri42 mice.

**Additional file 5: Table S4.** Excel spreadsheets that list the identity and spectral count data for the SDS-insoluble proteins identified from each model using SAINT score analysis. Each model is tabulated independently. The first tabs labeled “Raw spectra count, Removed duplicates, bait, prey, and inter” are the raw data exported from Scaffold and the intermediate steps of SAINT score calculations. SAINT scores were generated by SAINTexpress as described in Methods. The last 7 tabs are the identified proteins listed according to the genotypes. Any SDS-insoluble data with spectra counts less than 5 were removed, and only proteins with SAINT probability scores equal to or higher than 0.9 were counted as over-represented. This file contains the data used to generate Fig. 6.

**Additional file 6: Table S5.** List of the 40 proteins with the highest number of spectral counts relative to NTg controls in the forebrains of PrP.HuAβ/PS1 (Line 85) mice. Individual animal spectral counts are separated by a comma in the columns. The average NTg mouse spectral counts were calculated from two 20-month-old mice. All spectral count comparisons exhibited a G-test value of *p* < 0.05 and > 3 fold change in spectral count numbers between transgenics and NTg for every sample. Any protein with SAINT score lower than 0.90, calculated by comparison of transgenic to the aggregate 14 control NTg mice, was removed. SAINT fold is calculated by comparison of the spectrum counts of the three 20-month-old animals to all 14 NTg animals used across the whole study, regardless of age, batch of the LC-MS/MS run or doxycycline treatments. MW = molecular weight, PBS-S = PBS soluble fraction. The column labeled Compare to 2013 study provides notations on which proteins were previously identified in Table 1 of Xu et al. 2013 [42]. In that study, 28 proteins were identified as over-represented in SDS-insoluble fractions of 16-month-old PrP.HuAβ/PS1 (Line 85) mice. a = protein listed in Table 1 of [42]; b = identified in the 2013 study as having a higher level of spectral counts in SDS-insoluble fractions from PrP.HuAβ/PS1 mice but the values did not meet statistical criteria in that study (for example, if one sample from the transgenic had spectral counts for a given protein of < 5 then the protein did not meet criteria); c = identified only in the PBS-soluble fractions from NTg mice; d = not identified in any fraction in the 2013 study. Genes listed in red font were identified in the 2013 study and confirmed independently by immunoblotting. Gene names with an asterisk were also identified as among the 40 with the highest number of spectral counts in SDS-insoluble fractions from Tet.HuAβ mice (listed in Table S6). **Table S6.** List of the 40 proteins with the highest number of spectral counts relative to NTg controls in 2.5 and 13-month-old Tet.HuAβ mouse forebrains with and without Dox treatment. Spectral counts from each brain extract are separated by a comma in the columns. All spectral count comparisons exhibited a G-test value of *p* < 0.05 and > 3 fold change in spectral count numbers between transgenic and NTg mice for every sample. Any protein with SAINT score lower than 0.90 probability was removed. For the proteins listed here, the average spectral counts in 13-month old NTg controls were 0 or < 1 (see Additional files 3 and 4, Tables S2 and S3). SAINT fold is calculated by comparison of the spectrum counts of the transgenic animals to all 14 NTg animals used in this study, regardless of age, batch of LC-MS/MS run or doxycycline treatments. 1 week and 4 weeks indicate how long the mice were treated with Dox food to inhibit the APP transgene expression. No DOX indicated that these mice were not exposed to doxycycline food after they were weaned. The ages of the mice are noted above the columns of spectral count data. MW = molecular weight, PBS-S = PBS soluble fraction. The gene names with an asterisk identify proteins also found in the top 40 list for PrP.HuAβ/PS1 (Line 85) mice (listed in Table S5).
