# Supplementary material for: Diversity in Aβ deposit morphology and secondary proteome insolubility across models of Alzheimer-type amyloidosis
Source: Acta Neuropathol Commun. 2020 Apr 6;8:43. doi: 10.1186/s40478-020-00911-y (PMC7137436; doi:10.1186/s40478-020-00911-y)
Supplement: Supplementary file 1 — Additional file 1: Figure S1. The pathologic profile of the AD-amyloidosis models used in the study. All the images shown here are from mice that provided data for Table 2. The images shown are from tissues stained by the Campbell-Switzer silver (CS-Silver) method to detect all types of Aβ deposition, anti-Aβ antibodies 33.1.1, and 4G8 to detect Aβ, Thioflavin-S and Congo Red to detect cored deposits, anti-ubiquitin immunostaining to detect neuritic profiles, anti-GFAP immunostaining to detect astrogliosis, anti-Iba1 to identify microglia, and anti-Map 2 to show the distruption of neuropil by Aβ deposits. Images of Silver stains, Thioflavin-S, anti-ubiquitin, and GFAP are reproductions of images shown in Fig. 1 with digital enlargement. Low power images of the entire hemibrain were captured by scanning microscopy with an Aperio system. At least 3 animals were used for the staining and a representative section from 2 or 3 sections per animal is shown. a) Bri42Homo, b) Tet.Bri42, c) PrP.HuAβ/PS1, d) PrP.MoAβ/PS1, e) PrP.APPsi, f) Tet.MoAβ, g) Tet.HuAβ. Except for GFAP staining (amplification is 20x, scale bars 100 μm), all the other images were captured at 40x (scale bars 50 μm). Representative scale bars are provided. Figure S2. Venn diagram of the overlap of Tet.HuAβ and PrP.HuAβ/PS1 mice analyzed based upon LC-MS/MS analysis of SDS-insoluble proteins. These diagrams are based on the pair-wise statistical analysis combined with the SAINT scoring method of identifying proteins that were over-represented SDS-insoluble fractions from the brains of the Tet.HuAβ mice versus PrP.HuAβ/PS1 mice. Figure S3. Comparison of mass spectral data between instruments. The graph shows the relationship between spectral count data and protein identification data across two datasets generated with different instruments. The blue line graphs data obtained with a QStar instrument used in a study published in 2013 [42]. The red line graphs data obtained for the current study with a QE+ ins [file 40478_2020_911_MOESM1_ESM.pdf]

Figure S1

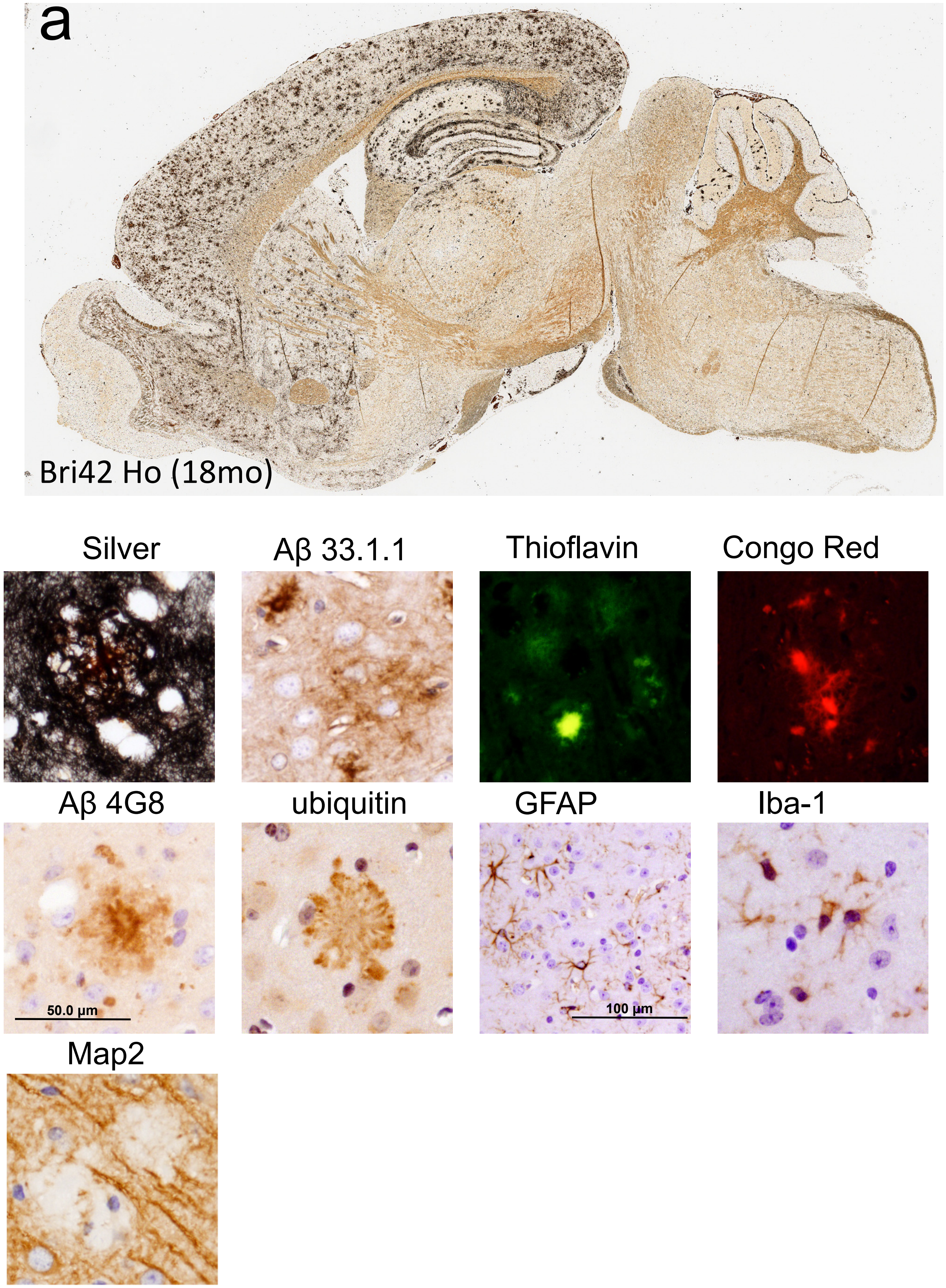
a
Bri42 Ho (18mo)
Silver
Aβ 33.1.1
Thioflavin
Congo Red
Aβ 4G8
ubiquitin
GFAP
Iba-1
50.0 µm
100 µm
Map2

Figure S1

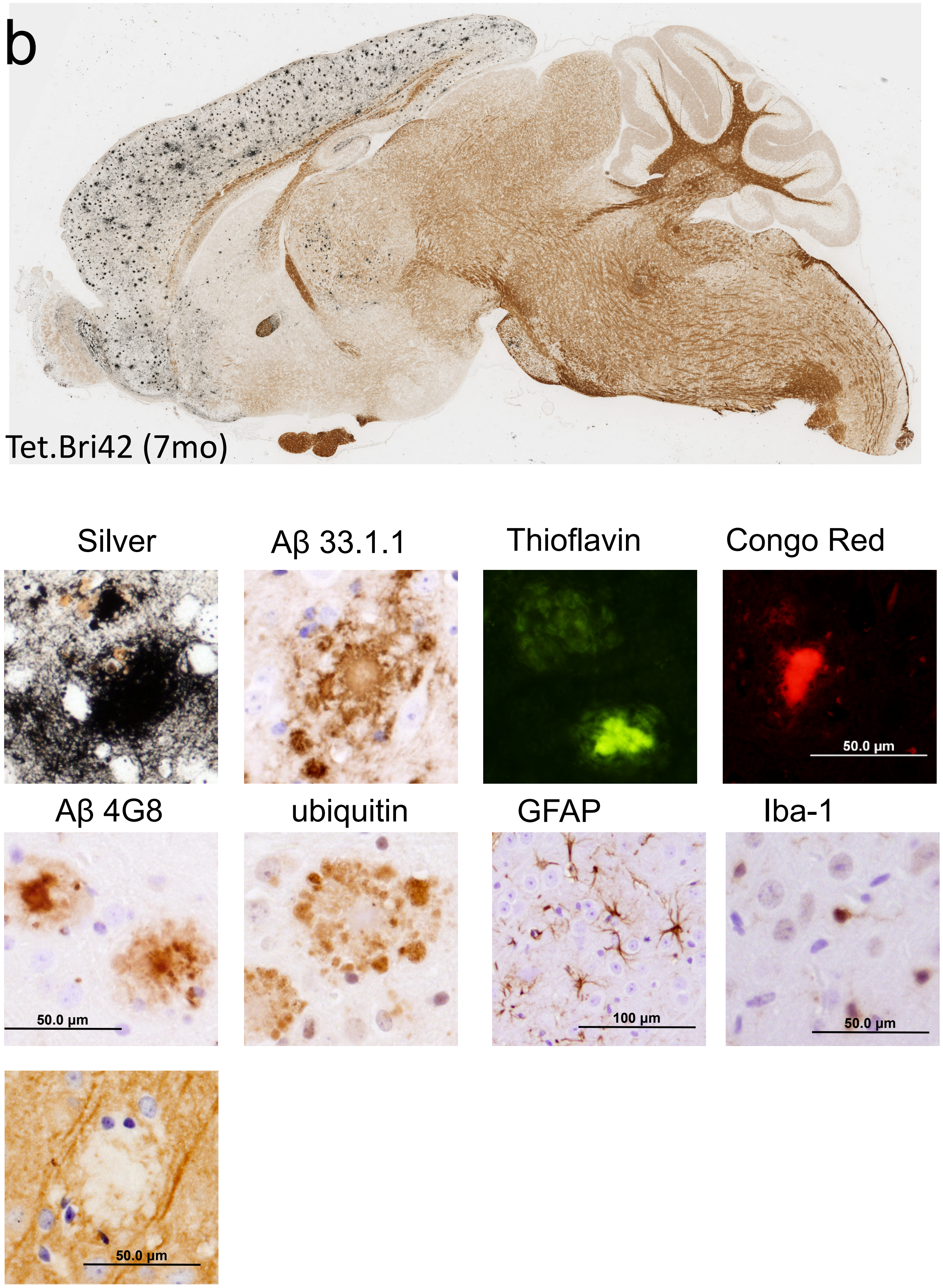
b
Tet.Bri42 (7mo)
Silver
Aβ 33.1.1
Thioflavin
Congo Red
50.0 µm
Aβ 4G8
ubiquitin
GFAP
Iba-1
50.0 µm
100 µm
50.0 µm
50.0 µm

Figure S1

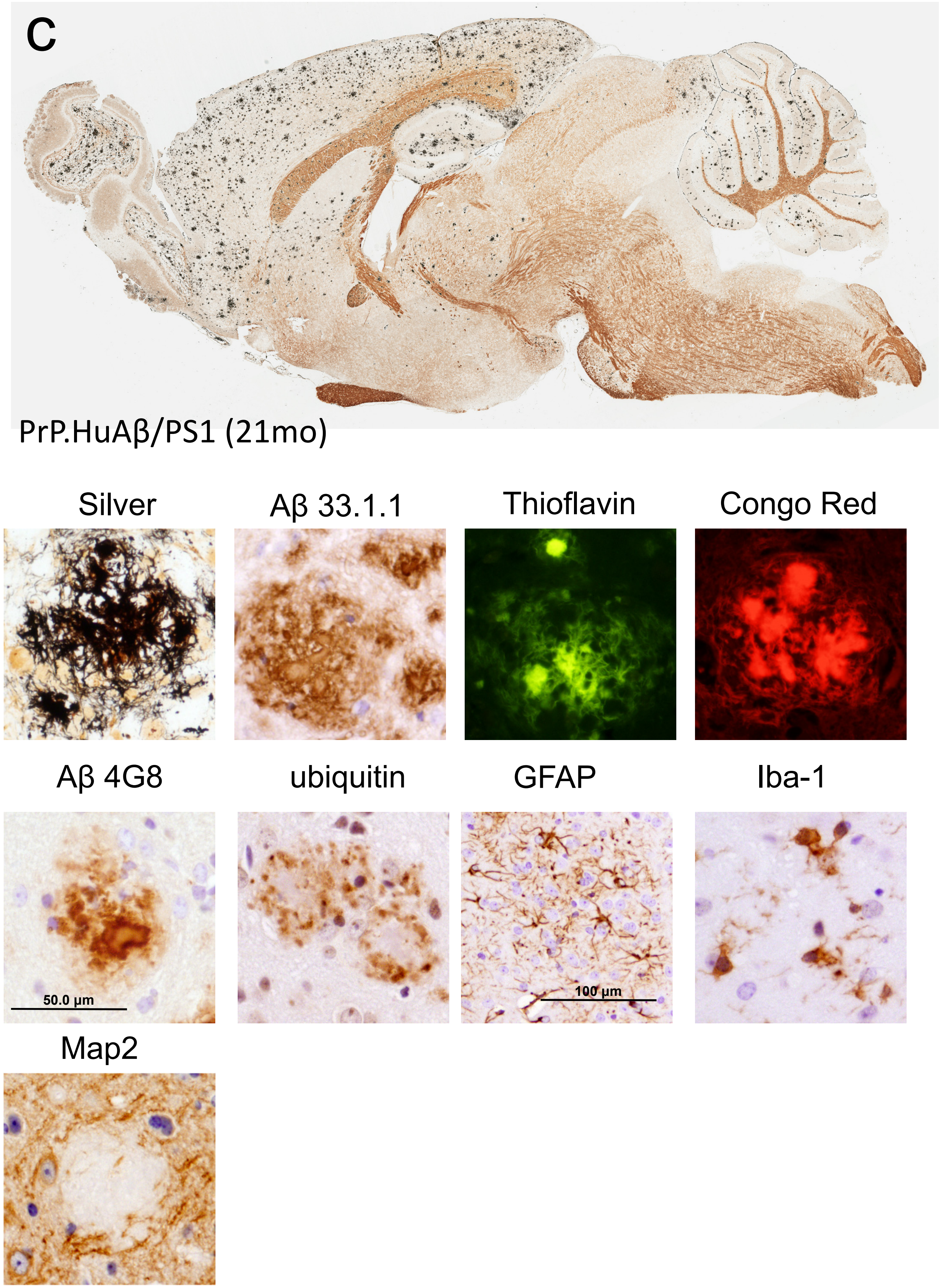
C
PrP.HuAβ/PS1 (21mo)
Silver
Aβ 33.1.1
Thioflavin
Congo Red
Aβ 4G8
ubiquitin
GFAP
Iba-1
50.0 µm
100 µm
Map2

Figure S1

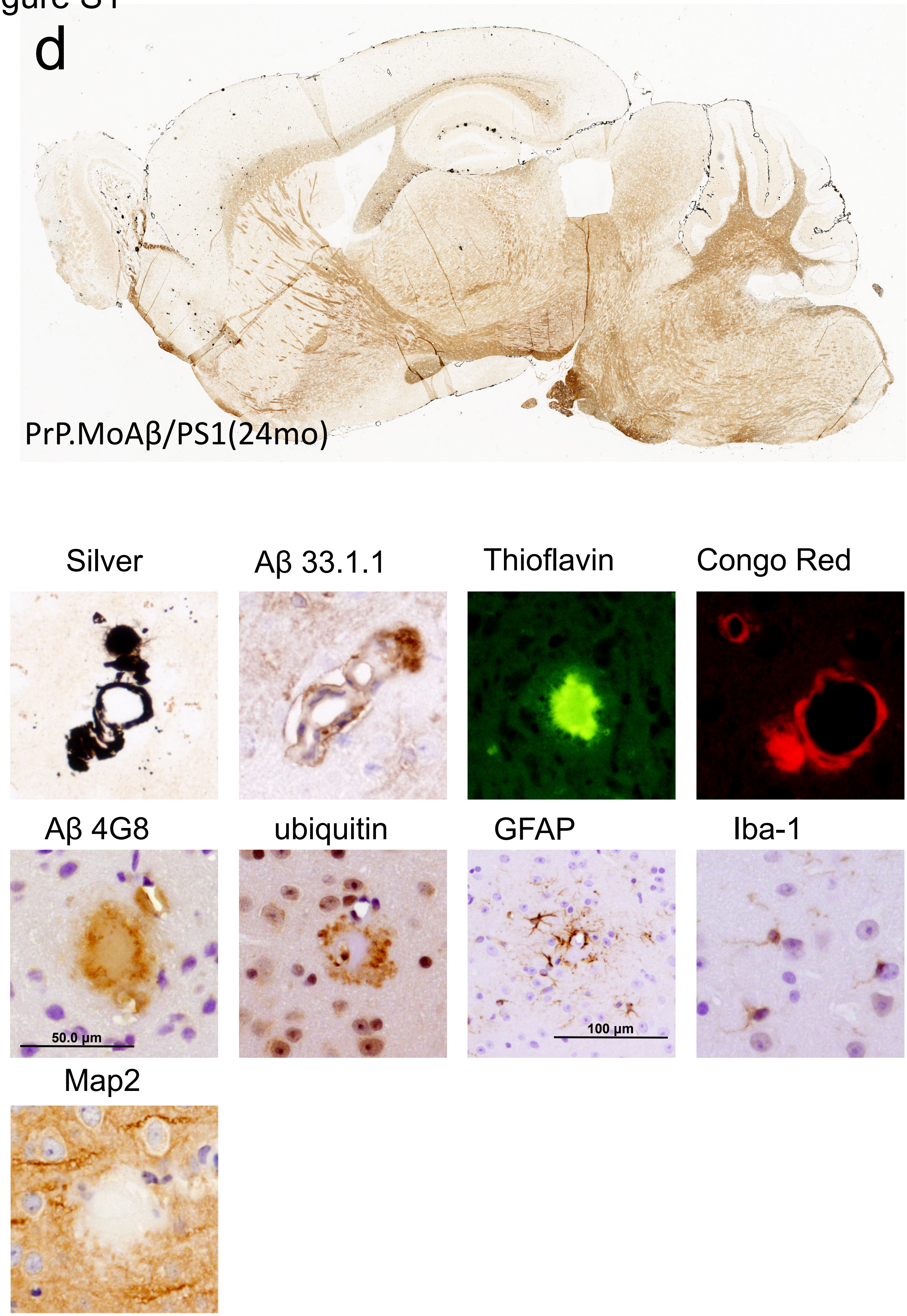

d
PrP.MoAβ/PS1(24mo)
Silver
Aβ 33.1.1
Thioflavin
Congo Red
Aβ 4G8
50.0 µm
ubiquitin
GFAP
100 µm
Iba-1
Map2

Figure S1

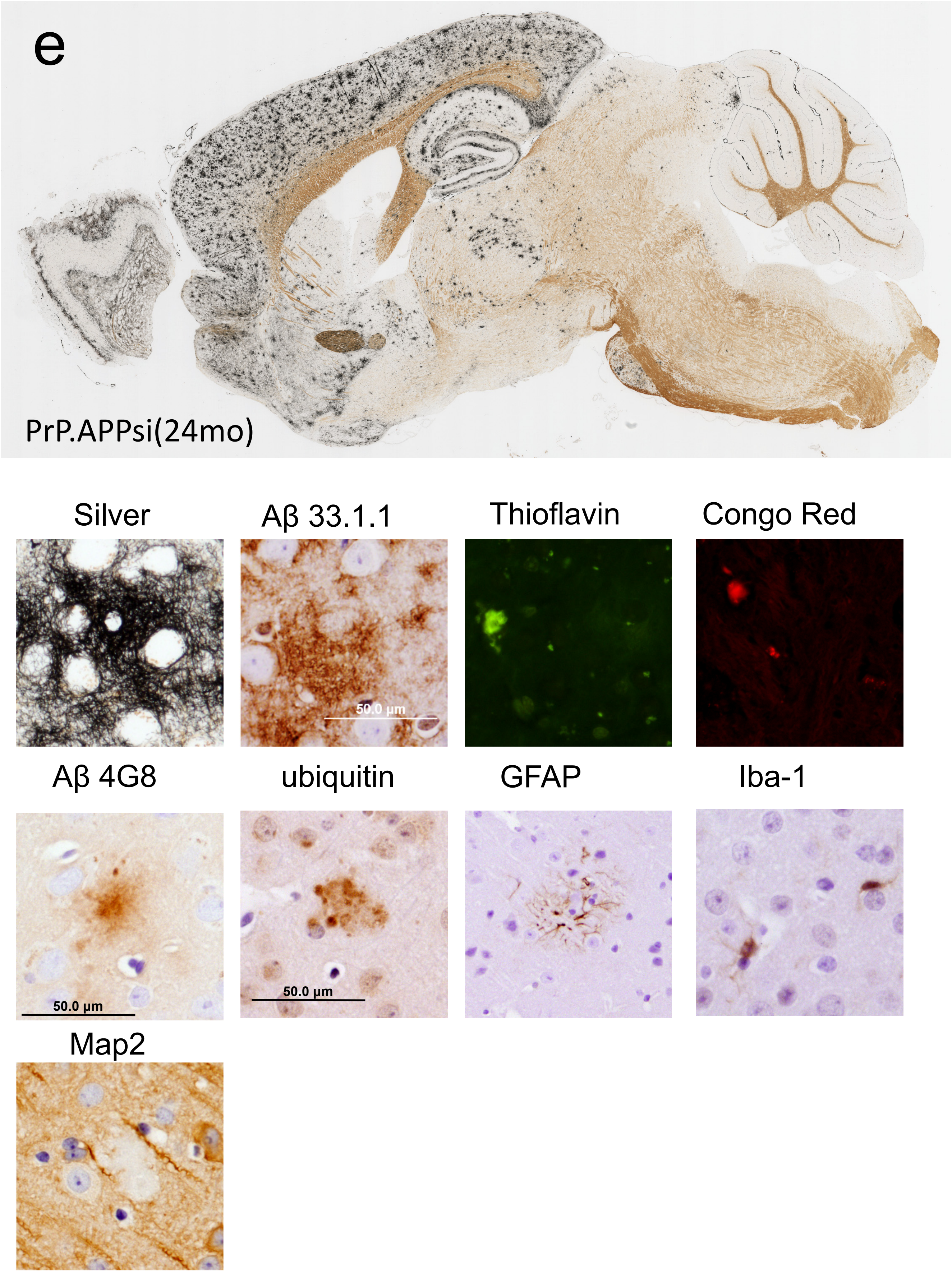

e
PrP.APPsi(24mo)
Silver
Aβ 33.1.1
Thioflavin
Congo Red
50.0 µm
Aβ 4G8
ubiquitin
GFAP
Iba-1
50.0 µm
50.0 µm
Map2

Figure S1

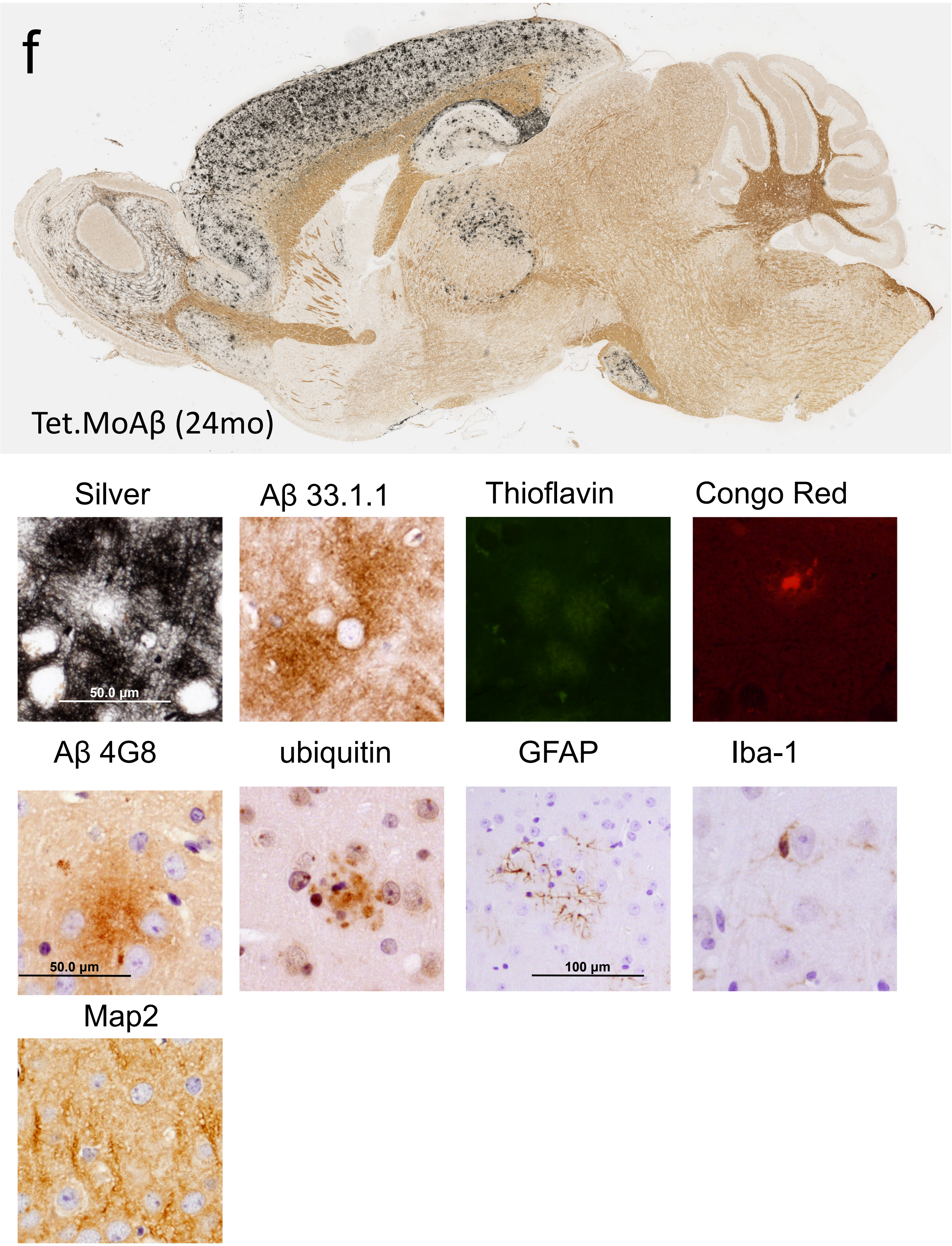

f
Tet.MoAβ (24mo)
Silver
Aβ 33.1.1
Thioflavin
Congo Red
50.0 µm
Aβ 4G8
ubiquitin
GFAP
Iba-1
50.0 µm
100 µm
Map2

Figure S1

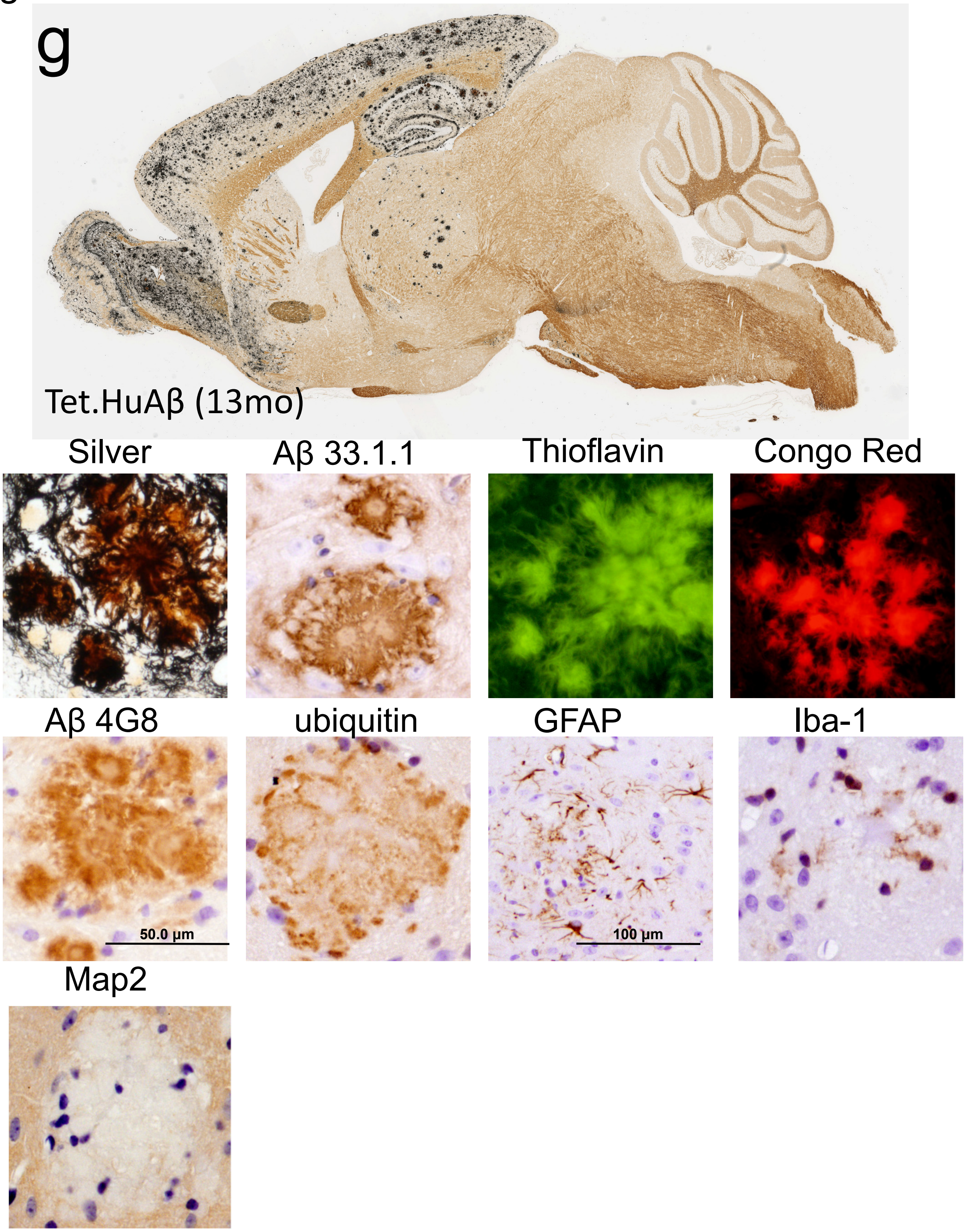

g
Tet.HuAβ (13mo)
Silver
Aβ 33.1.1
Thioflavin
Congo Red
Aβ 4G8
50.0 µm
ubiquitin
GFAP
100 µm
Iba-1
Map2

Figure S1

g - continued

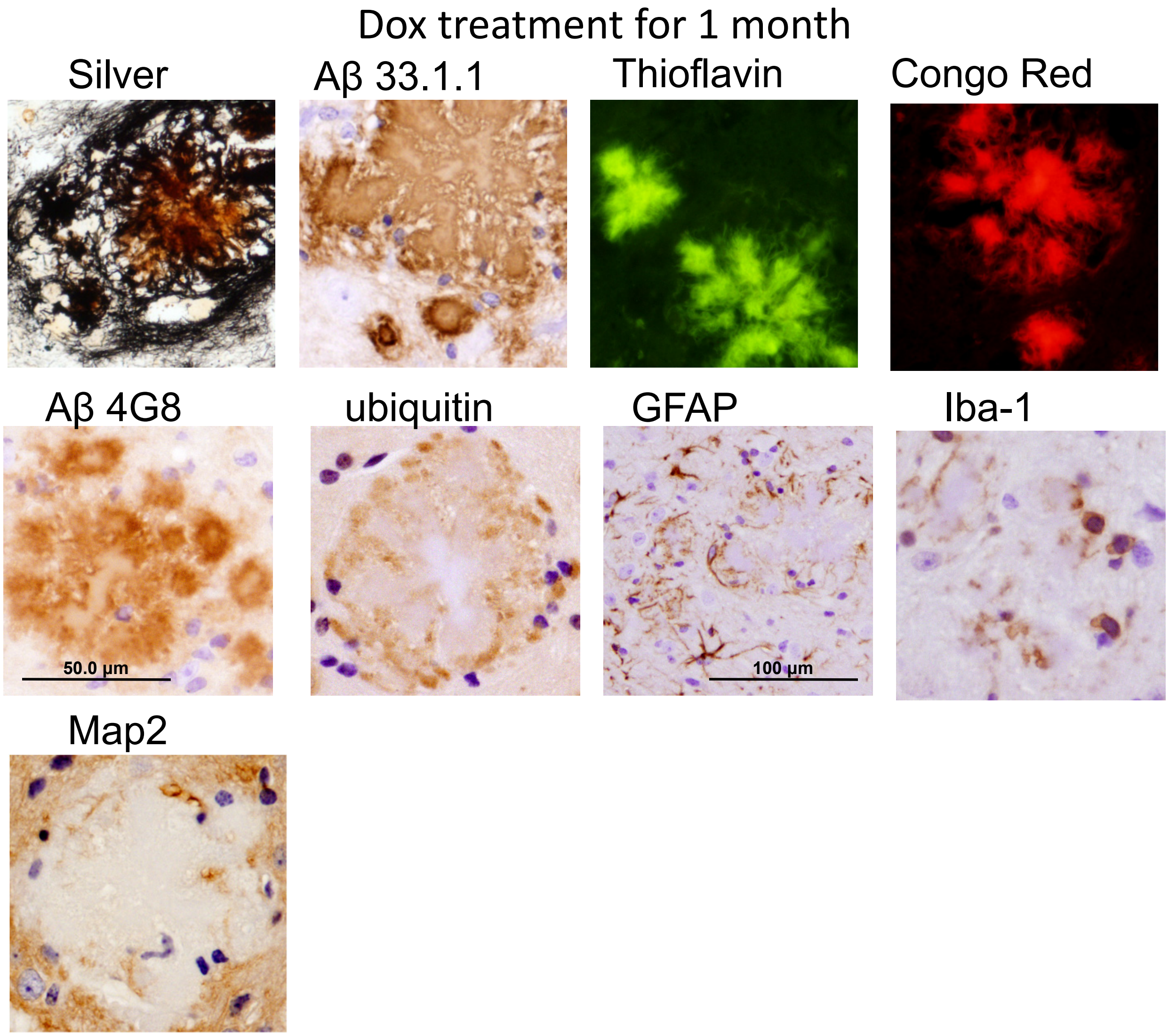

Figure S2

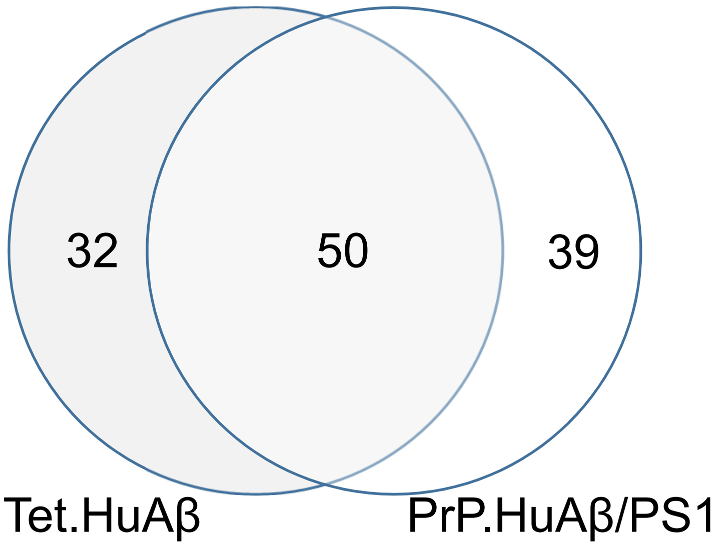

Figure S3

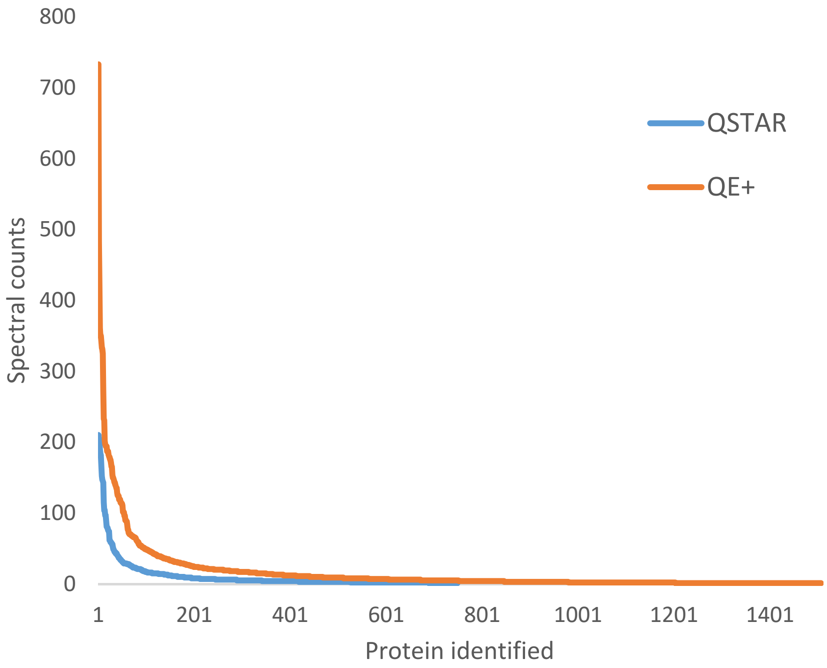

800
700
600
500
400
300
200
100
0
Spectral counts
1
201
401
601
801
1001
1201
1401
Protein identified
QSTAR
QE+
