# Supplementary material for: Diversity in Aβ deposit morphology and secondary proteome insolubility across models of Alzheimer-type amyloidosis
Source: Acta Neuropathol Commun. 2020 Apr 6;8:43. doi: 10.1186/s40478-020-00911-y (PMC7137436; doi:10.1186/s40478-020-00911-y)
Supplement: Supplementary file 6 — Additional file 6: Table S5. List of the 40 proteins with the highest number of spectral counts relative to NTg controls in the forebrains of PrP.HuAβ/PS1 (Line 85) mice. Individual animal spectral counts are separated by a comma in the columns. The average NTg mouse spectral counts were calculated from two 20-month-old mice. All spectral count comparisons exhibited a G-test value of p < 0.05 and > 3 fold change in spectral count numbers between transgenics and NTg for every sample. Any protein with SAINT score lower than 0.90, calculated by comparison of transgenic to the aggregate 14 control NTg mice, was removed. SAINT fold is calculated by comparison of the spectrum counts of the three 20-month-old animals to all 14 NTg animals used across the whole study, regardless of age, batch of the LC-MS/MS run or doxycycline treatments. MW = molecular weight, PBS-S = PBS soluble fraction. The column labeled Compare to 2013 study provides notations on which proteins were previously identified in Table 1 of Xu et al. 2013 [42]. In that study, 28 proteins were identified as over-represented in SDS-insoluble fractions of 16-month-old PrP.HuAβ/PS1 (Line 85) mice. a = protein listed in Table 1 of [42]; b = identified in the 2013 study as having a higher level of spectral counts in SDS-insoluble fractions from PrP.HuAβ/PS1 mice but the values did not meet statistical criteria in that study (for example, if one sample from the transgenic had spectral counts for a given protein of < 5 then the protein did not meet criteria); c = identified only in the PBS-soluble fractions from NTg mice; d = not identified in any fraction in the 2013 study. Genes listed in red font were identified in the 2013 study and confirmed independently by immunoblotting. Gene names with an asterisk were also identified as among the 40 with the highest number of spectral counts in SDS-insoluble fractions from Tet.HuAβ mice (listed in Table S6). Table S6. List of the 40 proteins with the highest number of spe [file 40478_2020_911_MOESM6_ESM.docx]

Table S5. The first 40 proteins ranked by spectral count abundance in the SDS-insoluble fractions from the brains of PrP.HuAβ/PS1 mice.

| **Gene** | **Protein** | **Accession Number** | **MW (KDa)** | **SDS-insoluble** | | | | | **PBS-S** | **SAINT Fold**  **(Tg vs. NTg)** | **Compare to**  **2013 study** | | | |
| --- | --- | --- | --- | --- | --- | --- | --- | --- | --- | --- | --- | --- | --- | --- |
|  |  |  |  | **NTg** | **PrP.HuAβ/PS1 transgenic mice (months)** | | | |  |  |  |  |  |  |
|  |  |  |  | **20** | **9** | **12** | **16** | **20** | **13** |  | **a** | **b** | **c** | **d** |
| Gapdh | Glyceraldehyde-3-phosphate dehydrogenase | S4R1W1_MOUSE | 36 | 45,34 | 63,75 | 97 | 120 | 160,175,153 | 156,150 | 5 | + | + |  |  |
| *Apoe | Apolipoprotein E | APOE_MOUSE | 36 | 3,1 | 80,73 | 89 | 130 | 125,139,187 | 11,15 | 54 | + | + |  |  |
| Dnm1 | Isoform 4 of Dynamin-1 | DYN1_MOUSE | 97 | 20,21 | 58,52 | 93 | 111 | 138,140,79 | 92,87 | 5 | + | + |  |  |
| *Ckb | Creatine kinase B-type | KCRB_MOUSE | 43 | 17,27 | 34,47 | 39 | 73 | 94,107,119 | 190,158 | 8 | + | + |  |  |
| Hspa8 | Heat shock cognate 71 kDa protein | Q3TRH3_MOUSE | 71 | 20,22 | 60,52 | 70 | 102 | 113,99,77 | 64,60 | 4 | + | + |  |  |
| Eno1 | Alpha-enolase | ENOA_MOUSE | 47 | 9,11 | 35,35 | 54 | 66 | 80,84,90 | 117,95 | 9 | + | + |  |  |
| *Stxbp1 | Syntaxin-binding protein 1 | STXB1_MOUSE | 68 | 16,11 | 48,49 | 58 | 75 | 88,85,45 | 45,49 | 7 | + | + |  |  |
| *Aldoa | Fructose-bisphosphate aldolase | A6ZI44_MOUSE | 45 | 9,11 | 15,17 | 34 | 42 | 72,67,46 | 106,97 | 9 | + | + |  |  |
| Ywhaz | 14-3-3 protein zeta/delta | 1433Z_MOUSE | 28 | 15,7 | 32,32 | 44 | 49 | 66,67,52 | 32,35 | 7 | + | + |  |  |
| *Uba1 | Ubiquitin-activating enzyme E1, Chr X | B9EHN0_MOUSE | 118 | 7,5 | 22,21 | 41 | 54 | 67,69,45 | 61,56 | 13 |  | + |  |  |
| *Hsp90aa1 | Heat shock protein HSP 90-alpha | HS90A_MOUSE | 85 | 2,6 | 21,17 | 49 | 55 | 56,66,37 | 102,87 | 10 | + | + |  |  |
| *Fasn | Fatty acid synthase | FAS_MOUSE | 272 | 0,1 | 8,3 | 28 | 30 | 56,61,30 | 53,50 | 14 |  | + |  |  |
| *App | Amyloid-beta protein-like protein long isoform | Q53ZT3_MOUSE | 87 | 5,0 | 21,20 | 23 | 78 | 44,40,62 | 4,19 | 38 |  | + |  |  |
| Ywhab | Tyrosine 3-monooxygenase/tryptophan 5-monooxygenase activation protein, beta polypeptide | A2A5N2_MOUSE | 28 | 9,3 | 22,25 | 33 | 42 | 50,51,45 | 26,24 | 8 | + | + |  |  |
| Tpi1 | Triosephosphate isomerase | TPIS_MOUSE | 32 | 6,6 | 30,29 | 41 | 45 | 53,52,40 | 31,34 | 10 |  | + |  |  |
| *Hsp90ab1 | Heat shock protein HSP 90-beta | HS90B_MOUSE | 83 | 2,6 | 18,15 | 42 | 48 | 52,54,35 | 108,94 | 8 | + | + |  |  |
| *Gpi | Glucose-6-phosphate isomerase | G6PI_MOUSE | 63 | 1,1 | 22,22 | 35 | 51 | 50,59,26 | 48,48 | 14 |  | + |  |  |
| Ywhag | 14-3-3 protein gamma | 1433G_MOUSE | 28 | 11,3 | 23,24 | 34 | 40 | 42,44,49 | 28,22 | 8 | + | + |  |  |
| Ywhaq | Tyrosine 3-monooxygenase/tryptophan 5-monooxygenase activation protein, theta polypeptide | A3KML3_MOUSE | 28 | 11,4 | 23,23 | 29 | 41 | 48,48,39 | 28,34 | 7 | + | + |  |  |
| Gdi1 | Rab GDP dissociation inhibitor alpha | GDIA_MOUSE | 51 | 3,1 | 22,21 | 22 | 41 | 53,56,25 | 46,42 | 13 | + | + |  |  |
| *Ppp2r1a | Serine/threonine-protein phosphatase 2A 65 kDa regulatory subunit A alpha isoform | 2AAA_MOUSE | 65 | 1,0 | 19,19 | 23 | 48 | 53,66,14 | 53,45 | 21 |  | + |  |  |
| *Pgk1 | Phosphoglycerate kinase 1 | PGK1_MOUSE | 45 | 6,2 | 22,21 | 30 | 48 | 52,48,33 | 47,46 | 8 | + | + |  |  |
| Ywhae | 14-3-3 protein epsilon | 1433E_MOUSE | 29 | 9,2 | 19,22 | 28 | 42 | 47,47,35 | 54,56 | 8 | + | + |  |  |
| *Pgam1 | Phosphoglycerate mutase 1 | PGAM1_MOUSE | 29 | 3,3 | 15,17 | 25 | 31 | 46,37,41 | 31,34 | 18 | + | + |  |  |
| *Eno2 | Gamma-enolase | Q3UJ20_MOUSE | 47 | 1,0 | 13,14 | 15 | 28 | 42,42,37 | 123,102 | 16 | + | + |  |  |
| Dnm3 | Dynamin-3 | E9QLL2_MOUSE | 97 | 7,7 | 14,13 | 23 | 33 | 40,45,29 | 41,33 | 5 |  | + |  |  |
| Hspa2 | Heat shock protein 70-2 | B7U582_MOUSE | 70 | 9,7 | 26,24 | 32 | 34 | 39,34,37 | 22,18 | 4 |  |  |  | + |
| *Atp6v1a | V-type proton ATPase catalytic subunit A | VATA_MOUSE | 68 | 0,4 | 9,9 | 26 | 31 | 39,42,28 | 51,49 | 10 |  | + |  |  |
| *Ywhah | 14-3-3 protein eta | 1433F_MOUSE | 28 | 9,0 | 21,17 | 28 | 34 | 37,39,31 | 26,22 | 9 | + | + |  |  |
| *Ppia | Peptidyl-prolyl cis-trans isomerase A | PPIA_MOUSE | 18 | 7,7 | 12,10 | 25 | 28 | 36,36,34 | 26,24 | 8 |  | + |  |  |
| *Mdh2 | Malate dehydrogenase, mitochondrial | MDHM_MOUSE | 36 | 4,6 | 21,17 | 31 | 39 | 42,36,24 | 25,24 | 6 |  | + |  |  |
| *Vcp | Transitional endoplasmic reticulum ATPase | TERA_MOUSE | 89 | 0,0 | 3,1 | 12 | 19 | 36,38,25 | 37,42 | 39 |  | + |  |  |
| Atp6v1b2 | V-type proton ATPase subunit B, brain isoform | VATB2_MOUSE | 57 | 1,5 | 8,11 | 20 | 25 | 34,38,24 | 48,40 | 9 |  | + |  |  |
| *Rap1gds1 | Protein Rap1gds1 | Q3TU36_MOUSE | 66 | 0,1 | 16,13 | 23 | 35 | 35,43,14 | 33,28 | 17 |  | + |  |  |
| Mdh1 | Malate dehydrogenase, cytoplasmic | MDHC_MOUSE | 37 | 4,2 | 15,14 | 21 | 28 | 35,33,21 | 20,24 | 13 |  | + |  |  |
| Pkm | Pyruvate kinase PKM | KPYM_MOUSE | 58 | 3,3 | 9,10 | 21 | 33 | 38,32,19 | 109,89 | 9 |  | + |  |  |
| *Napb | Beta-soluble NSF attachment protein | SNAB_MOUSE | 34 | 1,0 | 12,11 | 15 | 25 | 35,34,16 | 17,15 | 11 |  | + |  |  |
| Usp5 | Ubiquitin carboxyl-terminal hydrolase 5 | Q3U4W8_MOUSE | 93 | 1,1 | 5,5 | 15 | 20 | 26,32,18 | 34,28 | 22 |  | + |  |  |
| *Eef2 | Elongation factor 2 | EF2_MOUSE | 95 | 0,0 | 9,4 | 12 | 23 | 33,35,7 | 44,39 | 22 |  | + |  |  |
| *Tkt | Transketolase | TKT_MOUSE | 68 | 0,3 | 3,4 | 8 | 16 | 21,28,25 | 35,35 | 16 |  |  | + |  |
| **Total** |  |  |  |  |  |  |  |  |  |  | **20** | **38** | **1** | **1** |
| **Ratio (%)** |  |  |  |  |  |  |  |  |  |  | **50** | **95** | **3** | **3** |

Table S6. The first 40 proteins ranked by spectral count abundance in the SDS-insoluble fractions from the brains of Tet.HuAβmice.

| **Gene** | **Protein** | **Accession Number** | **MW**  **(KDa)** | **SDS-insoluble** | | | | **PBS-S** | **SAINT**  **fold** | **Non-specific binding** |
| --- | --- | --- | --- | --- | --- | --- | --- | --- | --- | --- |
|  |  |  |  | **No DOX** | | **1 week** | **4 weeks** |  |  |  |
|  |  |  |  | **2.5mo** | **13mo** | | | |  |  |
| *Ckb | Creatine kinase B-type | KCRB_MOUSE | 43 | 11,9,19 | 52,52,62,59 | 37,34,48 | 23,58,32, | 190,158 | 4.0 |  |
| *Fasn | Fatty acid synthase | FAS_MOUSE | 272 | 1,2,0 | 41,42,59,57 | 30,21,31 | 31,40,29, | 53,50 | 13.9 |  |
| *Apoe | Apolipoprotein E | APOE_MOUSE | 36 | 0,4,2 | 42,55,48,51 | 66,61,50 | 52,47,43, | 11,15 | 17.6 |  |
| *Stxbp1 | Syntaxin-binding protein 1 | STXB1_MOUSE | 68 | 9,11,15 | 40,42,43,50 | 28,27,41 | 31,47,30, | 45,49 | 4.1 |  |
| *App | Amyloid-beta protein-like protein long isoform | Q53ZT3_MOUSE | 87 | 1,2,1 | 27,64,32,50 | 38,44,29 | 35,45,33, | 4,19 | 33.6 |  |
| *Hsp90ab1 | Heat shock protein HSP 90-beta | HS90B_MOUSE | 83 | 6,7,6 | 33,36,47,43 | 29,26,36 | 32,39,28, | 108,94 | 7.0 |  |
| *Uba1 | Ubiquitin-activating enzyme E1, Chr X | B9EHN0_MOUSE | 118 | 1,8,4 | 33,38,41,41 | 36,26,36 | 31,36,30, | 61,56 | 8.2 |  |
| *Hsp90aa1 | Heat shock protein HSP 90-alpha | HS90A_MOUSE | 85 | 7,2,4 | 29,37,36,38 | 36,29,30 | 37,40,30, | 102,87 | 6.8 |  |
| *Pgk1 | Phosphoglycerate kinase 1 | PGK1_MOUSE | 45 | 5,3,3 | 27,31,31,32 | 35,31,31 | 30,28,25, | 47,46 | 5.3 |  |
| *Aldoa | Fructose-bisphosphate aldolase | A6ZI44_MOUSE | 45 | 2,1,1 | 23,32,31,34 | 21,18,27 | 27,27,19, | 106,97 | 4.5 |  |
| Synj1 | Synaptojanin-1 | D3Z656_MOUSE | 176 | 9,9,5 | 30,24,30,34 | 18,18,22 | 19,30,23, | 38,39 | 6.3 |  |
| *Gpi | Glucose-6-phosphate isomerase | G6PI_MOUSE | 63 | 2,2,0 | 20,27,29,31 | 26,23,23 | 22,23,22, | 48,48 | 8.5 |  |
| *Atp6v1a | V-type proton ATPase catalytic subunit A | VATA_MOUSE | 68 | 5,7,8 | 26,22,25,28 | 14,14,18 | 17,25,18, | 51,49 | 6.8 |  |
| *Ppp2r1a | Serine/threonine-protein phosphatase 2A 65 kDa regulatory subunit A alpha isoform | 2AAA_MOUSE | 65 | 1,2,6 | 24,24,27,25 | 20,21,19 | 19,26,20, | 53,45 | 11.7 |  |
| *Napb | Beta-soluble NSF attachment protein | SNAB_MOUSE | 34 | 5,5,2 | 23,26,23,27 | 22,19,23 | 22,27,23, | 17,15 | 9.4 |  |
| *Eno2 | Gamma-enolase | Q3UJ20_MOUSE | 47 | 2,0,2 | 14,23,29,25 | 15,18,25 | 18,24,17, | 123,102 | 8.9 |  |
| Dctn1 | Dynactin subunit 1 | E9Q3M3_MOUSE | 140 | 5,3,1 | 22,19,24,23 | 13,17,18 | 21,23,15, | 17,19 | 9.1 | + |
| *Rap1gds1 | Protein Rap1gds1 | Q3TU36_MOUSE | 66 | 1,3,1 | 18,20,26,23 | 15,13,21 | 13,20,16, | 33,28 | 11.7 |  |
| Gdi2 | Rab GDP dissociation inhibitor beta | Q3UC72_MOUSE | 57 | 0,1,1 | 13,17,28,28 | 12,13,26 | 18,25,12, | 40,36 | 10.0 |  |
| Gls | Glutaminase kidney isoform, mitochondrial | GLSK_MOUSE | 74 | 10,10,4 | 19,19,21,22 | 17,18,18 | 17,20,15, | 24,22 | 4.7 |  |
| *Ppia | Peptidyl-prolyl cis-trans isomerase A | PPIA_MOUSE | 18 | 4,1,2 | 17,16,23,25 | 12,15,21 | 17,25,16, | 26,24 | 4.7 |  |
| *Vcp | Transitional endoplasmic reticulum ATPase | TERA_MOUSE | 89 | 0,0,0 | 16,18,22,24 | 18,15,18 | 16,19,12, | 37,42 | 23.3 |  |
| *Pgam1 | Phosphoglycerate mutase 1 | PGAM1_MOUSE | 29 | 0,1,0 | 14,20,19,23 | 19,14,17 | 14,18,16, | 31,34 | 8.1 |  |
| *Eef2 | Elongation factor 2 | EF2_MOUSE | 95 | 0,2,1 | 13,16,23,22 | 16,10,19 | 17,20,15, | 44,39 | 16.2 |  |
| Acly | ATP-citrate synthase | Q3V117_MOUSE | 121 | 2,2,0 | 19,20,15,19 | 15,13,18 | 16,18,16, | 26,24 | 13.5 |  |
| Pygb | Glycogen phosphorylase, brain form | PYGB_MOUSE | 97 | 0,0,0 | 9,17,25,22 | 11,11,13 | 15,16,10, | 60,56 | 18.3 |  |
| Kif5c | Kinesin heavy chain isoform 5C | KIF5C_MOUSE | 109 | 2,3,0 | 15,20,17,20 | 15,9,11 | 15,13,17, | 22,23 | 11.0 |  |
| Hspa4l | Heat shock 70 kDa protein 4L | HS74L_MOUSE | 94 | 0,0,0 | 14,16,21,20 | 13,12,18 | 17,18,14, | 35,32 | 24.9 |  |
| Mapk1 | Mitogen-activated protein kinase 1 | MK01_MOUSE | 41 | 0,2,0 | 10,16,22,23 | 8,8,16 | 9,20,9, | 21,20 | 49.7 |  |
| Vps35 | Vacuolar protein sorting-associated protein 35 | Q3TJ43_MOUSE | 92 | 2,3,0 | 15,11,22,20 | 12,13,14 | 12,20,12, | 29,31 | 9.9 |  |
| Cct2 | Chaperonin subunit 2 (Beta), isoform CRA_a | Q542X7_MOUSE | 57 | 0,0,0 | 12,12,23,19 | 9,4,14 | 8,14,4, | 27,24 | 165.0 |  |
| Npepps | Puromycin-sensitive aminopeptidase | PSA_MOUSE | 103 | 0,0,0 | 14,15,16,21 | 17,13,18 | 13,21,13, | 29,29 | 23.1 |  |
| *Tkt | Transketolase | TKT_MOUSE | 68 | 0,1,0 | 12,16,18,20 | 12,9,18 | 11,16,11, | 35,35 | 10.5 |  |
| *Mdh2 | Malate dehydrogenase, mitochondrial | MDHM_MOUSE | 36 | 3,4,3 | 16,15,17,17 | 24,19,13 | 19,19,17, | 25,24 | 3.1 |  |
| Napa | Alpha-soluble NSF attachment protein | SNAA_MOUSE | 33 | 2,2,0 | 15,17,18,14 | 10,9,12 | 12,15,14, | 7,6 | 12.4 |  |
| Hspa4 | Heat shock 70 kDa protein 4 | Q3U2G2_MOUSE | 94 | 0,0,0 | 17,17,14,14 | 16,11,13 | 16,16,14, | 33,40 | 24.1 |  |
| Cand1 | Cullin-associated NEDD8-dissociated protein 1 | CAND1_MOUSE | 136 | 0,1,1 | 17,11,17,16 | 17,12,14 | 14,16,10, | 50,42 | 8.5 |  |
| Wdr1 | WD repeat domain 1 | Q3TJY2_MOUSE | 66 | 0,0,0 | 9,10,18,24 | 9,10,14 | 11,17,10, | 17,23 | 53.4 |  |
| *Ywhah | 14-3-3 protein eta | 1433F_MOUSE | 28 | 3,3,4 | 15,17,15,14 | 16,13,11 | 16,16,14, | 26,22 | 3.9 |  |
| Aldoc | Fructose-bisphosphate aldolase C | ALDOC_MOUSE | 39 | 2,2,0 | 10,20,14,15 | 12,9,9 | 18,19,14, | 58,54 | 4.2 |  |
